# Ecology and evolution of pyrazines in insects

**DOI:** 10.1002/brv.70160

**Published:** 2026-03-20

**Authors:** Zowi Oudendijk, Niklas Wahlberg, Johanna Mappes

**Affiliations:** ^1^ Department of Organismal & Evolutionary Biology, Faculty of Biological and Environmental Sciences University of Helsinki Helsinki Finland; ^2^ Biodiversity Unit, Department of Biology Lund University Lund Sweden

**Keywords:** pyrazine, insect, evolution, function, synthesis, allomone, pheromone

## Abstract

Chemical communication is the oldest and most widespread form of signalling among and within organisms. Among the many compounds involved in such communication, pyrazines – nitrogen‐containing heterocyclic molecules – are especially intriguing due to their widespread occurrence across the tree of life, from bacteria and fungi to insects and mammals. In this review, we focus on the role of pyrazines in insects, where they mediate both intra‐ and interspecific communication. Social insects, particularly within the order Hymenoptera, exhibit a greater diversity and frequency of pyrazine use compared to solitary species and other insect orders. This diversity may be associated with the complexity of communication systems required by eusociality. Pheromonal pyrazines predominantly feature alkyl and alkenyl group substituents, whereas allomonal pyrazines more often feature methoxy groups. Pyrazines have been identified in seven insect orders. Hemimetabolous insects, such as Phasmatodea, Orthoptera, and Hemiptera typically produce alkyl‐substituted pyrazines, with some Hemiptera also producing methoxy variants. Methoxy‐substituted pyrazines are absent in Hymenoptera but present in Coleoptera and Lepidoptera, where they serve as both pheromones and allomones. In Diptera, pyrazines are only known from a few species, and have alkyl or alkenyl substituents. Pyrazines are mainly associated with adult stages, suggesting a predominant role in later‐life communication but more research is needed on early life stages. Current evidence suggests that pyrazine biosynthesis may be carried out by microbial symbionts. To understand fully the evolutionary origins and ecological functions of pyrazines in insects, comprehensive surveys across taxa and life stages alongside functional studies are essential.

## I. INTRODUCTION

Animals communicate with each other using a variety of signals, including visual, acoustic, tactile and chemical stimuli. Communication between organisms facilitates the exchange of information, leading to alteration in behaviours and traits. Chemical communication represents the most prevalent and oldest signalling method within and between organisms (Steiger, Schmitt & Schaefer, 2010; Speed *et al*., 2012). It manifests in diverse forms, ranging from intraspecies communication to broader interspecies communication (Abd El‐Ghany, 2019). This diversity and complexity have long been a source of inspiration for scientists, who have dedicated significant efforts to studying the evolution of these interactions (Stökl & Steiger, 2017; Mayorga‐Martino *et al*., 2025). The functions of chemical communications manifest in diverse forms, including pheromones, which serve as sexual attractants (Buchinger & Li, 2023; Groot *et al*., 2024), or induce aggregation (Mitaka *et al*., 2020) within species. Allomones, too, play a significant role in communication, often exhibiting a defensive function (Nathalia *et al*., 2024). A notable illustration of this phenomenon is the defensive strategies employed by insects, with predation pressure shown to influence the evolution of defensive chemicals (Sugiura, 2020; Kannan, Galizia & Nouvian, 2022). The properties of these chemical compounds are selected to maximise their performance and functions, for example, when they are used in communication to predators that prey are not edible or to maximise dispersal for alarm pheromones. Volatile compounds are frequently utilised due to their rapid diffusion through the air, in contrast to less‐volatile compounds (Silva‐Junior *et al*., 2018; Yang *et al*., 2025). The chemicals employed in communication are selected based on the properties of the compounds themselves, and they are synthesised or obtained from external sources (often food) to fulfil their function (Kannan *et al*., 2022; Nathalia *et al*., 2024). The selective optimisation of chemical compounds for enhanced performance has recently gained increasing attention (Beran *et al*., 2019). In this review, we aim to explore the evolution and ecology of the chemical group pyrazines.

**Fig. 1:**
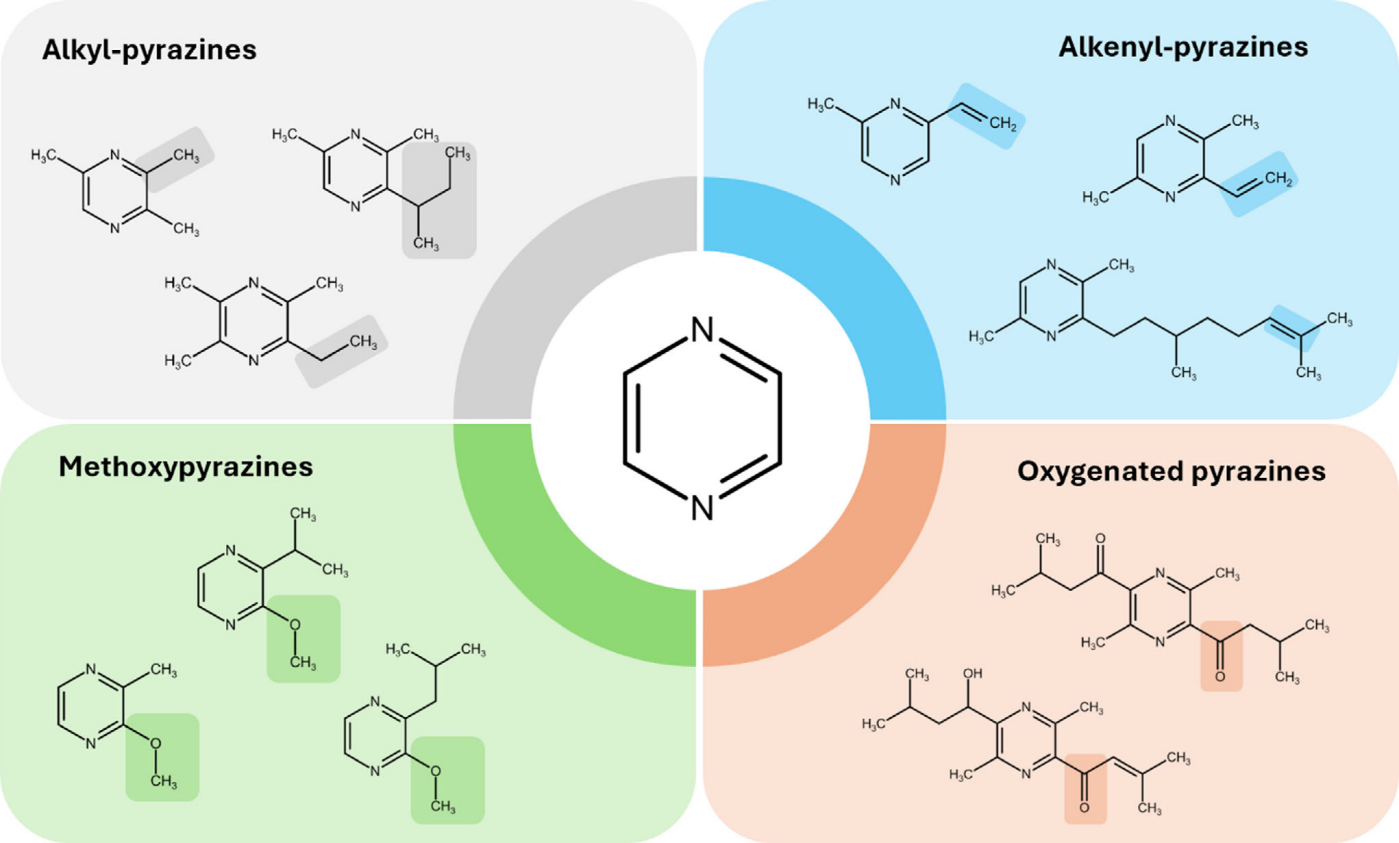
The general structure of pyrazines (central diagram), and examples of pyrazines with substituents reported to be present in insects: alkyl‐, alkenyl‐, methoxy‐, and oxygenated pyrazines (outer panels). The examples provided for each category are, from left to right: alkyl‐pyrazines – 2,5‐dimethyl‐3‐methylpyrazine, 2‐ethyl‐3,5,6‐trimethylpyrazine, and 2,6‐dimethyl‐3‐*sec*‐butylpyrazine; alkenyl‐pyrazines – 2‐methyl‐6‐vinylpyrazine, 2,5‐dimethyl‐3‐cirtonellylpyrazine and 2,5‐dimenthyl‐3‐vinylpyrazine; methoxypyrazines – 2‐methoxy‐3‐methylpyrazine, 2‐methoxy‐3‐isopropylpyrazine, and 2‐methoxy‐3‐isobutylpyrazine; oxygenated pyrazines – 2,5‐dimethyl‐3‐(1‐hydroxyisopentyl)‐6‐(1‐oxoisopent‐2‐enyl)‐pyrazine and 2,5‐dimethyl‐3‐(1‐oxoisopentyl)‐6‐(1‐oxoisopent‐2‐enyl)‐pyrazine.

Pyrazine is a two‐nitrogen‐containing monocyclic aromatic ring molecule of diazines orienting in a 1,4‐*para* position (Fig. 1). Its structural orientation provides greater stability than other isomers because the position of nitrogen atoms creates an electron‐withdrawing effect (Ong, Liu & Tay, 2017). Derivatives of pyrazine are found in combination with four possible substituents at one or more of the four‐ring carbon atoms (Fig. 1). The substituents of pyrazine affect its properties by altering its polarity, intermolecular forces, and volatility (Müller & Rappert, 2010; Zamolo & Wüst, 2023). These derivatives are likely to reflect the communication functions of pyrazines present in different organisms.

Pyrazines have been identified in a wide array of natural environments and are produced by numerous taxa, including bacteria (Rajini *et al*., 2011), plants (Zamolo & Wüst, 2023), fungi (Kłosowski, Mikulski & Pielech‐Przybylska, 2021), vertebrates (Mattina, Pignatello & Swihart, 1991; Müller‐Schwarze & Houlihan, 1991; Osada, Miyazono & Kashiwayanagi, 2014) and invertebrates (Moore, Brown & Rothschild, 1990; Burdfield‐Steel *et al*., 2018). The majority of pyrazines occurring in nature are found in low quantities (Müller & Rappert, 2010), and different taxa produce different pyrazine compounds (Brophy, 1989; Moore *et al*., 1990; Zamolo & Wüst, 2023), facilitating the different functions of these compounds. In addition to their natural occurrence, pyrazines have applications in agriculture and the food industry, including the production of wines, vegetables and pesticides (Zamolo & Wüst, 2023; Ren *et al*., 2024). The functions of synthetic pyrazines include antibacterial, anticancer and antiviral properties (Seliem *et al*., 2021). The presence of pyrazines contributes significantly to the aroma of various food items, including plant‐derived products such as cocoa, wine, and coffee, as well as meat and pastry products (Maga, 1992). The prevalence of pyrazines in dietary sources is noteworthy, particularly in light of a recent study that revealed the evolution of an olfactory receptor in mammals that specifically recognises pyrazine‐based food odours (Marcinek *et al*., 2021). Pyrazines are thought to be a key component in alarm signals in nature (Woolfson & Rothschild, 1997), but the evolution and ecological roles of pyrazines in animal communication have not been extensively discussed. This is primarily due to the fact that the majority of studies that have examined the presence of pyrazines in animals have focused on their synthesis and functions (Brophy, 1989; Moore *et al*., 1990). Herein, we seek to broaden the existing body of knowledge by providing a comprehensive overview of pyrazines in Insecta, one of the most diverse groups of taxa.

## II. PYRAZINES IN INSECTS

Insects interact in complex ways through chemical communication (Ali & Morgan, 1990), particularly the social insects (Leonhardt *et al*., 2016). Among insects, pyrazines were initially identified in ponerine ants (Formicidae; Hymenoptera), where they function as alarm pheromones (Wheeler & Blum, 1973). Subsequent advancements in analytical technologies led to the identification of a diverse array of pyrazines in insects (Cai, Koziel & O'Neal, 2007; Khashaveh *et al*., 2025). In a comprehensive review, Brophy (1989) explored the origin, synthesis and function of pyrazines in insects. This review notably omitted discussion of the ecological roles and evolution of pyrazines across diverse insect species. Using the substantial body of knowledge accumulated since Brophy's (1989) formative work, we review the literature on pyrazines in insects, including their detection, functions, and ecological roles. We also explore the evolution of pyrazines in insects, together providing a comprehensive overview of the current state of knowledge in this field.

### (1) Occurrence and diversity of pyrazines in insects

A comprehensive search was conducted to identify publications on pyrazines in insects. We searched for the key words ‘pyrazine*’ and ‘insect’ across all fields of the *Web of Science* database. We also included the names of each insect order as search terms. Literature associated with pyrazine semiochemicals derived from other sources, such as plants, that alter insect behaviour was excluded from consideration; thus, our focus is exclusively on cases involving the presence of pyrazines within insects.

**Fig. 2:**
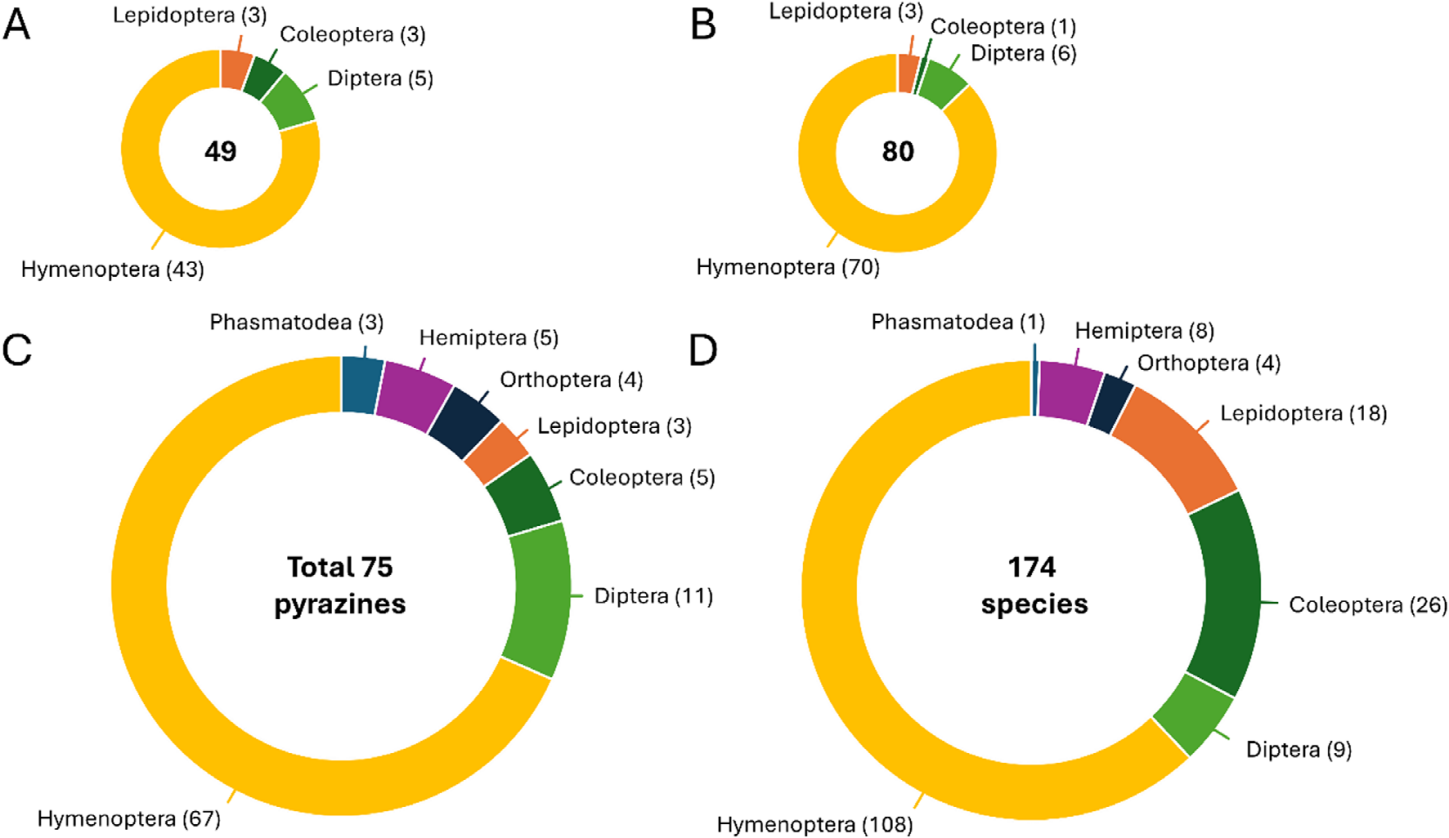
The proportion of insect species for which pyrazine compounds have been reported by Brophy (1989) (A, B) and by this study (C, D). The left doughnuts (A and C) represent the number of pyrazine compounds, and those on the right (B and D) represent the number of species found to carry pyrazines in each order.

A comprehensive list of all pyrazines detected within insects is presented in Table 1. A total of 75 different pyrazines have been identified in insects (see Table S1 for nomenclature, and Fig. S1 for chemical structures), covering seven different insect orders and 174 species (Fig. 2). The majority were found in Hymenoptera, mainly in ants. By contrast, pyrazines have been reported from only a limited number of species from Phasmatodea and Orthoptera. This disparity is likely due to less research on these orders, with Hymenoptera more extensively studied due to their complex social interactions.

**Table 1:** List of pyrazines detected within each insect species. See Table S1 for chemical names of numbered compounds. Classification of the species is listed up to subfamily level.

Order Superfamily Family Subfamily	Species	Compound	Reference
Phasmatodea			
Phyllioidea			
Phylliidae			
Phylliinae	*Phyllium westwoodii*	12,16,17	Dossey *et al*. (2009)
Orthoptera			
Acridoidea			
Acrididae			
Oedipodinae	*Locusta migratoria manilensis*	3	Shi *et al*. (2011)
Pyrgomorphidae			
Pyrgomorphinae	*Poekilocerus bufonius*	73	Moore *et al*. (1990)
Tettigoniidea			
Conocephalinae	*Vestria* sp.	1,3,4	Nickle *et al*. (1996)
Pseudophyllinae	*Pterophylla beltrani*	73	Torres‐Castillo *et al*. (2018)
Hemiptera			
Aphidoidea			
Aphidae			
Aphidinae	*Megoura viciae*	3	Leroy *et al*. (2012)
Cercoidea			
Aphrophoridae	*Lepyronia coleoptrata*	5	Körner (2006)
Aphrophoridae	*Philaenus spumarius*	5	Körner (2006)
Cercopidae	*Cercopis vulnerata*	5,73	Moore *et al*. (1990); Körner (2006)
Cercopidae	*Haematoloma dorsatum*	5	Körner (2006)
Membracoidea			
Membracidae	*Centrotus cornutus*	5	Körner (2006)
Pentatomoidea			
Pentatomidae			
Pentatomiae	*Murgantia histrionica*	73	Aldrich *et al*. (1996)
Reduvioidea			
Reduviidae			
Triatominae	*Triatoma dimidiata*	73,74,75	May‐Concha *et al*. (2015)
Lepidoptera			
Noctuoidea			
Erebidae			
Arctiinae	*Amata* sp.	72,73	Rothschild *et al*. (1984); Moore *et al*. (1990)
Arctiinae	*Arctia caja*	73,74	Moore *et al*. (1990)
Arctiinae	*Arctia plantaginis*	73,74	Rojas *et al*. (2017); Burdfield‐Steel *et al*. (2018)
Arctiinae	*Euplagia quadripunctaria*	73,74	Moore *et al*. (1990)
Arctiinae	*Tyria jacobaeae*	73	Moore *et al*. (1990)
Lymantriinae	*Leucoma salicis*	72	Aldrich *et al*. (1997)
Lymantriinae	*Lymantria dispar*	74	Aldrich *et al*. (1997)
Papilionoidea			
Nymphalidae			
Danainae	*Danaus plexippus*	72,73,74	Rothschild *et al*. (1984); Moore *et al*. (1990)
Heliconiinae	*Actinote pelleria*	73	Moore *et al*. (1990)
Heliconiinae	*Dryas iulia*	72,73,74	Moore *et al*. (1990)
Heliconiinae	*Heliconius atthis*	73,74	Moore *et al*. (1990)
Heliconiinae	*Heliconius charithonia*	72,73,74	Moore *et al*. (1990)
Heliconiinae	*Heliconius melpomene*	72,73,74	Schulz *et al*. (2008)
Papilionidae			
Papilioninae	*Battus polydamas*	73,74	Moore *et al*. (1990)
Papilioninae	*Papilio rumanzovia*	73,74	Moore *et al*. (1990)
Papilioninae	*Zerynthia polyxena*	73	Moore *et al*. (1990)
Zygaenoidea			
Zygaenidae			
Procridinae	*Pollanisus* sp.	73	Moore *et al*. (1990)
Zygaeninae	*Zygaena lonicerae*	73,74	Rothschild *et al*. (1984); Moore *et al*. (1990)
Diptera			
Tephritoidea			
Tephritidae			
Trypetinae	*Anastrepha curvicauda*	41,55	Chuman *et al*. (1987); Robledo *et al*. (2014)
Trypetinae	*Anastrepha fraterculus*	3,4,5,14	Lima *et al*. (2001)
Trypetinae	*Anastrepha serpentina*	3,4,25	Robacker *et al*. (2009)
Dacinae	*Bactrocera cucumis*	4,23	Kitching *et al*. (1989)
Dacinae	*Bactrocera cucurbitae*	1,4,5,22,23	Baker *et al*. (1982); Hadapad *et al*. (2016)
Dacinae	*Bactrocera dorsalis*	4,6,22,23	Perkins *et al*. (1990); Ren *et al*. (2021)
Dacinae	*Bactrocera zonata*	4,5,22,23	Levi‐Zada *et al*. (2020)
Dacinae	*Bactrocera occipitalis*	5	Perkins *et al*. (1990)
Dacinae	*Ceratitis capitata*	6	Baker *et al*. (1985)
Coleoptera			
Chrysomelloidea			
Chrysomellidae			
Clytrinae	*Labidostomis lusitanica*	72	López *et al*. (2023)
Coccinelloidea			
Coccinellidae			
Coccidulinae	*Rodatus boucardi*	72	Moore *et al*. (1990)
Coccinellinae	*Adalia bipunctata*	72,73,74	Moore *et al*. (1990); Susset *et al*. (2013)
Coccinellinae	*Coccinella septempunctata*	72,73,74	Moore *et al*. (1990); Kögel *et al*. (2012)
Coccinellinae	*Coccinella transversalis*	72,73	Moore *et al*. (1990)
Coccinellinae	*Harmonia axyridis*	72,73,74,75	Moore *et al*. (1990); Cai *et al*. (2007); Kögel *et al*. (2012); Schmidtberg *et al*. (2019)
Coccinellinae	*Harmonia conformis*	73	Moore *et al*. (1990)
Coccinellinae	*Hippodamia convergens*	72,73,74	Cudjoe *et al*. (2005); Wheeler & Cardé (2013)
Coccinellinae	*Illeis* sp.	73	Moore *et al*. (1990)
Coccinellinae	*Micraspis frenata*	73	Moore *et al*. (1990)
Epilachninae	*Epilachna vigintisexpunctata*	72,73,74	Moore *et al*. (1990)
Scyminiae	*Epilachna cucurbitae*	72	Moore *et al*. (1990)
Endomychidae	*Eumorphus tetraspilotus*	73	Moore *et al*. (1990)
Elateroidea			
Cantharidae			
Cantharinae	*Rhagonycha fulva*	73	Moore *et al*. (1990)
Lampyridae			
Photurinae	*Photuris trivittata*	72,73,74	Vencl *et al*. (2016)
Lycidae			
Lycinae	*Calopteron reticulatum*	72	Eisner *et al*. (2008)
Lycinae	*Calopteron terminale*	72	Eisner *et al*. (2008)
Lycinae	*Lycus arizonensis*	72	Eisner *et al*. (2008)
Lycinae	*Lycus fernandezi*	72	Eisner *et al*. (2008)
Lycinae	*Lycus fulvellus*	72	Eisner *et al*. (2008)
Lycinae	*Lycus loripes*	72	Eisner *et al*. (2008)
Lycinae	*Lycus sanguinipennis*	72	Eisner *et al*. (2008)
Metriorrhynchinae	*Metriorrhynchus rhipidius*	71,72,73	Moore & Brown (1981); Moore *et al*. (1990)
Tenebrionoidea			
Meloidae			
Nemognathinae	*Palaestra foveicollis*	73	Moore *et al*. (1990)
Nemognathinae	*Zonitis lutea*	72,73	Moore *et al*. (1990)
Oedemeridae	*Pseudolycus haemopterus*	73	Moore *et al*. (1990)
Hymenoptera			
Apoidea			
Apidae			
Nomadinae	*Epeolus cruciger*	5,12,16,17	Tengö *et al*. (1982)
Nomadinae	*Epeolus variegatus*	12,16,17	Tengö *et al*. (1982)
Crabronidae			
Bembicinae	*Argogorytes fargeii*	17	Borg‐Karlson & Tengö (1980)
Bembicinae	*Argogorytes mystaceus*	17	Borg‐Karlson & Tengö (1980)
Bembicinae	*Bicyrtes ventralis*	14,17	Wheeler *et al*. (1982)
Bembicinae	*Nysson spinosus*	17	Borg‐Karlson & Tengö (1980)
Crabroninae	*Tachytes guatemalensis*	14,17	Wheeler *et al*. (1982)
Philanthinae	*Philanthus triangulum*	7,17	Borg‐Karlson & Tengö (1980)
Sphecidae			
Ammophilinae	*Ammophila fernaldi*	17	Duffield *et al*. (1981)
Ammophilinae	*Ammophila nigricans*	17	Duffield *et al*. (1981)
Ammophilinae	*Ammophila procera*	17	Duffield *et al*. (1981)
Ammophilinae	*Ammophila urnaria*	8,15	Duffield *et al*. (1981)
Ammophilinae	*Eremnophila aureonotata*	14,17	Wheeler *et al*. (1982)
Thynnoidea			
Thynnidae			
Thynninae	*Macrothynnus insignis*	17	Bohman & Peakall (2014)
Thynninae	*Zaspilothynnus nigripes*	17,65	Bohman *et al*. (2012); Bohman & Peakall (2014)
Thynninae	*Zaspilothynnus rugicollis*	17	Bohman *et al*. (2014)
Thynninae	*Zaspilothynnus seductor*	17	Bohman *et al*. (2014)
Tiphioidea			
Tiphiidae			
Tiphiiinae	*Tiphia* sp.	17	Wheeler *et al*. (1982)
Vespoidea			
Vespidae			
Eumeninae	*Ancistrocerus antilope*	9	Hefetz & Batra (1980)
Eumeninae	*Ancistrocerus campestris*	15,17	Wheeler *et al*. (1982)
Eumeninae	*Eumenes fraternus*	17,18	Hefetz & Batra (1980); Wheeler *et al*. (1982)
Eumeninae	*Euodynerus fuscus*	15,17	Wheeler *et al*. (1982)
Eumeninae	*Leptochilus acolhuus*	14,17	Wheeler *et al*. (1982)
Eumeninae	*Monobia quadridens*	20	Wheeler *et al*. (1982)
Eumeninae	*Pachodynerus erynnis*	7,8	Wheeler *et al*. (1982)
Eumeninae	*Parancistrocerus fulvipes*	7,15,17,18	Wheeler *et al*. (1982)
Eumeninae	*Parancistrocerus perennis*	15,17	Wheeler *et al*. (1982)
Eumeninae	*Parancistrocerus rufovestis*	15,17	Wheeler *et al*. (1982)
Eumeninae	*Parancistrocerus* sp.	15,17	Wheeler *et al*. (1982)
Eumeninae	*Pseudodynerus quadrisectus*	17	Hefetz & Batra (1980)
Eumeninae	*Stenodynerus floridanus*	7,8	Wheeler *et al*. (1982)
Eumeninae	*Stenodynerus fulvipes*	9,12,17	Hefetz & Batra (1980)
Polistinae	*Polybioides raphigastra*	3,4,5,22,23	Sledge *et al*. (1999)
Stenogastrinae	*Parischnogaster mellyi*	3,4	Dani *et al*. (1998)
Formicoidea			
Formicidae			
Dolichoderinae	*Dolichoderus clarkii*	17	Brophy (1989)
Dolichoderinae	*Iridomyrmex nitidus*	17	Brophy (1989)
Dolichoderinae	*Iridomyrmex purpureus*	5,7,14	Cavill *et al*. (1984)
Dolichoderinae	*Iridomyrmex rufoniger*	8	Brophy (1989)
Dolichoderinae	*Linepithema humile*	7,18,42,43	Cavill & Houghton (1974)
Ectatomminae	*Ectatomma* sp.	5,7,12,16,19	Morgan *et al*. (1999)
Ectatomminae	*Rhytidoponera aciculata*	17	Brophy *et al*. (1983)
Ectatomminae	*Rhytidoponera metallica*	16,17,44,47–54	Brophy *et al*. (1981); Tecle *et al*. (1987)
Ectatomminae	*Rhytidoponera victoriae*	12,16,17	Brophy (1989)
Formicinae	*Calomyrmex* sp.	12,16,17	Brown & Moore (1979)
Formicinae	*Notoncus ectatommoides*	17	Brophy *et al*. (1982)
Formicinae	*Paratrechina longicornis*	2,4	Morgan *et al*. (2005)
Myrmicinae	*Acromyrmex octospinosus*	5,6	Cross *et al*. (1982)
Myrmicinae	*Aphaenogaster rudis*	45,46	Wheeler *et al*. (1982)
Myrmicinae	*Atta bisphaerica*	5	Mosquera & Oliveira (1990)
Myrmicinae	*Atta cephalotes*	5	Evershed & Morgan (1983)
Myrmicinae	*Atta sexdens rubropilosa*	3,5	Cross *et al*. (1979); Evershed & Morgan (1983); Silva‐Junior *et al*. (2018)
Myrmicinae	*Atta sexdens sexdens*	3,4,5	Evershed & Morgan (1983); Billen *et al*. (1992); Morgan *et al*. (2013)
Myrmicinae	*Daceton armigerum*	3,4,5	Morgan *et al*. (1992)
Myrmicinae	*Eutetramorium mocquerysi*	9,37,38	Tentschert *et al*. (2000)
Myrmicinae	*Manica rubida*	1,3,4,5	Attygalle *et al*. (1986)
Myrmicinae	*Messor arenarius*	3,4,5	Cruz‐López *et al*. (2006)
Myrmicinae	*Messor bouvieri*	5	Jackson *et al*. (1989)
Myrmicinae	*Messor mediorubra*	3,4,5	Cruz‐López *et al*. (2006)
Myrmicinae	*Messor pergandei*	3,4	Plowes *et al*. (2014)
Myrmicinae	*Messor rugosus*	5	Cruz‐López *et al*. (2006)
Myrmicinae	*Myrmica lobicornis*	5	Evershed *et al*. (1982)
Myrmicinae	*Myrmica rubra*	5	Evershed *et al*. (1982)
Myrmicinae	*Myrmica ruginodis*	5	Evershed *et al*. (1982)
Myrmicinae	*Myrmica rugulosa*	5	Evershed *et al*. (1982)
Myrmicinae	*Myrmica sabuleti*	5	Evershed *et al*. (1982)
Myrmicinae	*Myrmica scabrinodis*	5	Evershed *et al*. (1982)
Myrmicinae	*Myrmica schencki*	5	Evershed *et al*. (1982)
Myrmicinae	*Myrmica* sp.	5	Jackson *et al*. (1991)
Myrmicinae	*Myrmica sulcinodis*	5	Evershed *et al*. (1982)
Myrmicinae	*Myrmecia gulosa*	7	Brophy & Nelson (1985)
Myrmicinae	*Pheidole pallidula*	5	Ali *et al*. (1988, 2007)
Myrmicinae	*Pheidole sinaitica*	26,27,28	Ali *et al*. (2007)
Myrmicinae	*Pogonomyrmex barbatus*	3,4,5	Hölldobler *et al*. (2001)
Myrmicinae	*Pogonomyrmex maricopa*	3,4,5	Hölldobler *et al*. (2001)
Myrmicinae	*Pogonomyrmex occidentalis*	3,4,5	Hölldobler *et al*. (2001)
Myrmicinae	*Pogonomyrmex rugosus*	3,4,5	Hölldobler *et al*. (2001)
Myrmicinae	*Pogonomyrmex vermiculatus*	3,4,5	Torres‐Contreras *et al*. (2007)
Myrmicinae	*Solenopsis geminata*	5	Hu *et al*. (2018)
Myrmicinae	*Solenopsis invicta*	5,6	Vander Meer *et al*. (2010); Guan *et al*. (2014); Choi & Vander Meer (2015); Sun *et al*. (2017); Hu *et al*. (2018); Li *et al*. (2019)
Myrmicinae	*Solenopsis richteri*	5	Hu *et al*. (2018)
Myrmicinae	*Tetramorium caespitum*	3,5	Attygalle & Morgan (1983)
Myrmicinae	*Tetramorium impurum*	1,3,4,5	Attygalle *et al*. (1986)
Myrmicinae	*Tetramorium meridionale*	3,4,5	Jackson *et al*. (1990)
Myrmicinae	*Wasmannia auropunctata*	16,36	Showalter *et al*. (2010); Yu *et al*. (2014)
Ponerinae	*Anochetus sedilloti*	10,11,12,13,15,19	Longhurst *et al*. (1978)
Ponerinae	*Austroponera castanea*	17,24,44,56–64	Fales *et al*. (1984, 1988)
Ponerinae	*Austroponera castaneicolor*	17,24,44,56–64	Fales *et al*. (1984, 1988)
Ponerinae	*Brachyponera sennaarensis*	4,15,20	Longhurst *et al*. (1978); Nikbakhtzadeh *et al*. (2009, 2009)
Ponerinae	*Diacamma indicum*	4	Morgan *et al*. (2003)
Ponerinae	*Dinoponera australis*	7,12,14,16, 17,56,57,62,66	Oldham *et al*. (1994)
Ponerinae	*Dinoponera grandis*	7,17	Hermann *et al*. (1984)
Ponerinae	*Euponera* sp.	17	Brophy (1989)
Ponerinae	*Hypoponera opacior*	17	Duffield *et al*. (1976)
Ponerinae	*Leptogenys chinensis*	17	Fales *et al*. (1992)
Ponerinae	*Leptogenys kitteli*	17,24	Fales *et al*. (1992)
Ponerinae	*Leptogenys processionalis*	10	Fales *et al*. (1992)
Ponerinae	*Odontomachus bauri*	5,7,8,14,15,16,17,20,21,29,30,32,39,40	Morgan *et al*. (1999); Xu *et al*. (2018)
Ponerinae	*Odontomachus brunneus*	6,8,15,20	Wheeler & Blum (1973)
Ponerinae	*Odontomachus chelifer*	6,8,15,17,20,21	Xu *et al*. (2018)
Ponerinae	*Odontomachus clarus*	20	Wheeler & Blum (1973)
Ponerinae	*Odontomachus erythrocephalus*	17,21,39	Xu *et al*. (2018)
Ponerinae	*Odontomachus hastatus*	17	Wheeler & Blum (1973)
Ponerinae	*Odontomachus ruginodis*	15,17,20,21,39	Xu *et al*. (2018)
Ponerinae	*Odontomachus* sp.	8,15,20,21	Wheeler & Blum (1973)
Ponerinae	*Odontomachus troglodytes*	6,15,20,21,42	Longhurst *et al*. (1978)
Ponerinae	*Odontoponera transversa*	7,12,17,19	Morgan *et al*. (1999)
Ponerinae	*Pachycondyla indica*	5,12,62	Morgan *et al*. (1999)
Ponerinae	*Pachycondyla obscuricornis*	3,4,5	Morgan *et al*. (1999, 2003)
Ponerinae	*Pachycondyla striata*	5,7,12,14,17,19,30–35,62,67–70	Morgan *et al*. (1999, 2003)
Ponerinae	*Ponera pennsylvanica*	17	Duffield *et al*. (1976)
Ponerinae	*Streblognathus aethiopicus*	15,20,21	Jones *et al*. (1998)

### (2) Chemical substituents of pyrazines in insects

The predominant substitution group of pyrazine compounds found in insects are alkyl‐pyrazines (40 pyrazine compounds, with a single‐bonded carbon chain structure from the core pyrazine) (Fig. 1), followed by alkenyl‐pyrazine groups (18 pyrazines with a double‐bonded carbon chain), methoxypyrazines (5 pyrazines with a methoxy group), and oxygenated pyrazines (12 pyrazines with an oxygen‐bonded structure in the compound). The classification of these compounds in Table S1 is based on the pattern of substitutions on the core pyrazine structure (Fig. 1). These substituents affect chemical properties (Zamolo & Wüst, 2023) including volatility, which is an important property of allelochemicals and pheromones in insects for communication.

## III. INSECT ORDER‐SPECIFIC PYRAZINES

**Fig. 3:**
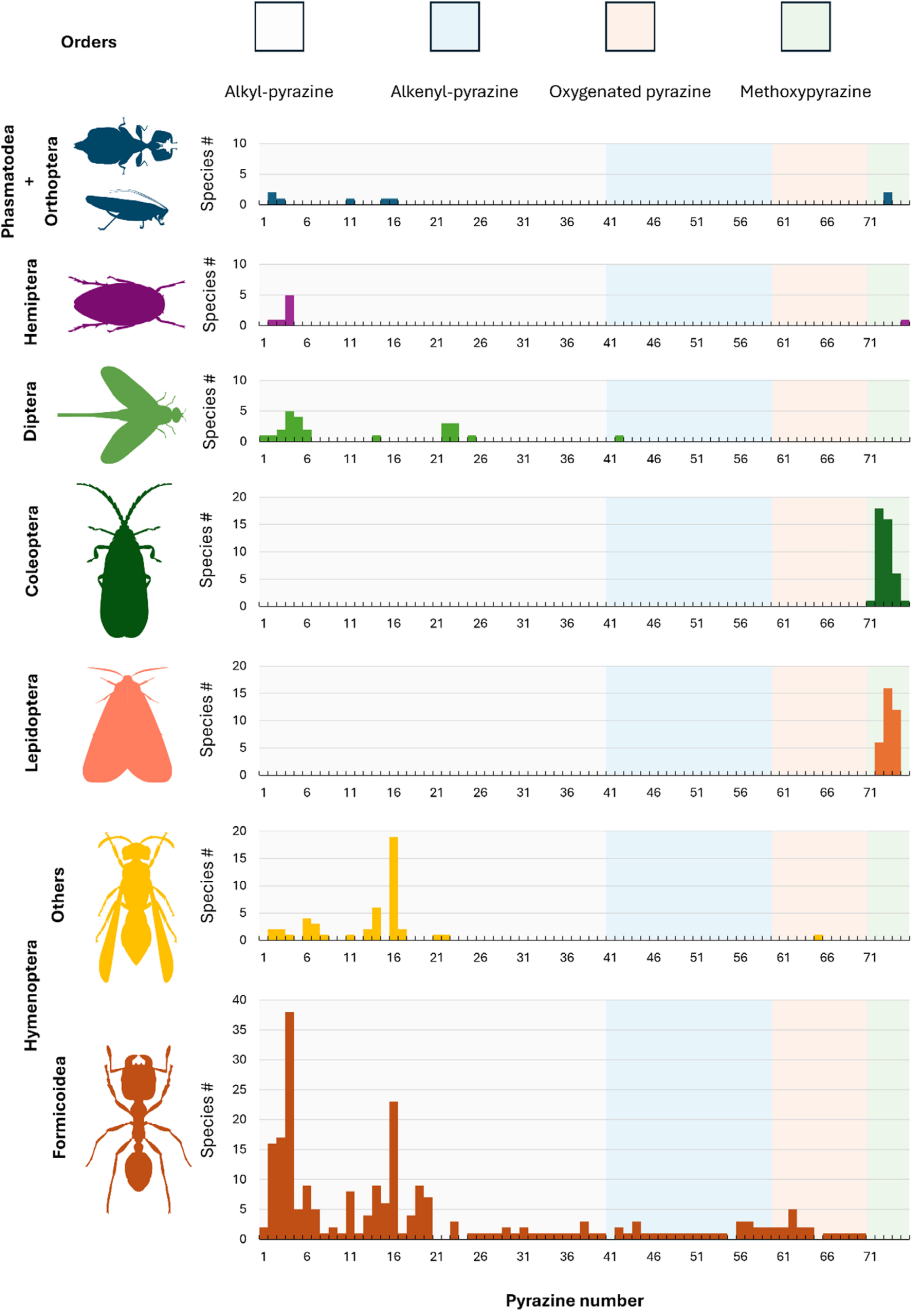
Summary of the pyrazines detected in different orders of insects. The background of the bar graph is shaded according to the different substituent groups (see key and Fig. 1). The height of each bar represents the number of species in which each pyrazine has been found. The numbers on the *x*‐axis represent the reference pyrazine numbers (see Table S1 for chemical names).

In this section we describe the presence of pyrazines in species belonging to each order in which they have been reported, their diversity and ecological roles (see Tables 1, S1 and Fig. 3).

### (1) Phasmatodea

The Phasmatodea, a group of insects that includes the stick and leaf insects, comprises approximately 3,500 described species. These species are notable for their camouflage, mimicking the colours and textures of surrounding plants. Additionally, they have developed chemical defences against predators, involving the release of irritating substances in the form of a spray from the prothoracic exocrine gland when they are disturbed (Eisner *et al*., 1997). Among the numerous species in which the chemical composition of the spray has been examined, only one species, *Phyllium westwoodii*, has been found to release pyrazines. The spray contains a mixture of three different pyrazines, 2,5‐dimethyl‐3‐isobutylpyrazine (12), 2,5‐dimethyl‐3‐(2‐methylbutyl)‐pyrazine (16), and 2,5‐dimethyl‐3‐isopentylpyrazine (17) (Dossey *et al*., 2009), together with other compounds including alpha and beta glucose. The release of these defensive pyrazines has been associated with a tube‐like gland in the prothorax (Niekampf *et al*., 2024). While deterrence of predators has been examined for the pyrazines detected from this species, further research is necessary to determine the full scope of their functions. By contrast, the use of 2,5‐dimethyl‐3‐(2‐methylbutyl)‐pyrazine (16) and 2,5‐dimethyl‐3‐isopentylpyrazine (17) have been identified as alarm pheromones in various hymenopteran species (Duffield, Blum & Wheeler, 1976; Showalter *et al*., 2010). The presence of pyrazines in *P. westwoodii* has only been documented for females (Dossey *et al*., 2009), and their absence in other Phasmatodea led Dossey *et al*. (2009) to propose that this feature is apomorphic.

### (2) Orthoptera

In Orthoptera, pyrazines have been identified in only a few species (Table 1). A single methoxypyrazine was reported for *Poekilocerus bufonius* and *Pterophylla beltrani*: 2‐methoxy‐3‐*sec*‐butylpyrazine (73). This compound was detected from whole‐body samples (Moore *et al*., 1990; Torres‐Castillo *et al*., 2018). The role of such compounds as defensive chemicals has been well documented in other orders, including Coleoptera and Lepidoptera, and is considered likely in Orthoptera as well. Further research is necessary to investigate the behavioural effects of these compounds. In a migratory species, *Locusta migratoria manilensis*, 2,5‐dimethylpyrazine (3) was detected in faecal samples from both sexes (Shi *et al*., 2011) and stimulated aggregation behaviour only among adults. Conversely, nymphs of the same species exhibited no response to this pyrazine (Shi *et al*., 2011). In a Neotropical species from the genus *Vestria*, a secretion released from the odoriferous gland of the abdomen of both sexes contained methylpyrazine (1), 2,5‐dimethylpyrazine (3), and 2,5‐dimethyl‐3‐methylpyrazine (4) (Nickle *et al*., 1996). The primary predators of these Neotropical orthopterans are primates, and these pyrazines may function defensively, although this remains to be explored.

### (3) Hemiptera

Eight species of Hemiptera have been observed to produce pyrazines in their secretions when subjected to stress (Table 1). For example, when *Triatoma dimidiata* (Reduviidae) is disturbed, it releases 2‐methoxy‐3‐*sec*‐butylpyrazine (73), 2‐methoxy‐3‐isobutylpyrazine (74), and 2,5‐dimethyl‐3‐methoxypyrazine (75) among other compounds (May‐Concha *et al*., 2015). The release of these compounds was observed from both sexes and in third‐stage nymphs when disturbed. These pyrazines may therefore function either as alarm pheromones or in defence against predators. The pyrazines were not found in the fluids released from exocrine glands, while secreted volatiles from the exocrine gland did induce avoidance behaviour by others (May‐Concha *et al*., 2015). The pyrazine 2,5‐dimethyl‐3‐methoxypyrazine (75) has only been documented in one other insect, the harlequin ladybird *Harmonia axyridis* (Coleoptera) (Cai *et al*., 2007).

Vetch aphids (*Megoura viciae*; Aphididae) produce honeydews that contain 2,5‐dimethylpyrazine (3) (Leroy *et al*., 2012). This pyrazine was released together with other kairomones produced by the aphid that attracted the predator *Harmonia axyridis*. A study specifically examining methoxypyrazines found no evidence for their presence in two aphid species, *Aphis nerii* and *A. persicae* (Moore *et al*., 1990). Nevertheless, further investigation may be worthwhile, particularly on their excreted honeydews. The methoxypyrazine 2‐methoxy‐3‐*sec*‐butylpyrazine (73) has been identified in two additional hemipterans: in the reflex‐bleed fluid of the harlequin bug (*Murgantia histrionica*; Pentatomoidea) (Aldrich *et al*., 1996) and in a whole‐body sample from red‐black froghopper (*Cercopis vulnerata*; Cercoidea) (Moore *et al*., 1990). In another study on *C. vulnerata*, an alkyl‐pyrazine, 2,5‐dimethyl‐3‐ethylpyrazine (5) was detected in the reflex‐bleed fluid of both sexes, while 2‐methoxy‐3‐*sec*‐butylpyrazine (73) was not detected (Körner, 2006). In several other Cercoidea species (*Lepyronia coleoptrata*, *Philaenus spumarius*, and *Haematoloma dorsatum*) and in *Centrotus cornutus* (Membracoidea), 2,5‐dimethyl‐3‐ethylpyrazine (5) was also detected from reflex‐bleed fluid (Körner, 2006).

### (4) Diptera

In Diptera, pyrazines have been identified within only a restricted taxonomic group of fruit flies, specifically those belonging to the Tephritidae family, encompassing *Anastrepha*, *Bactrocera*, and *Ceratitis*. Despite this limited taxonomic distribution of pyrazines within the Diptera, the diversity of these compounds is notable (Table 1). The majority of the pyrazines detected in tephritids are produced by males, and some of these are known to function as sex pheromones (Table 2). Virgin male papaya fruit flies (*Anastrepha curvicauda*) produce two vinylpyrazines, 2‐methyl‐6‐vinylpyrazine (41) and 2,5‐dimethyl‐3‐vinylpyrazine (55); this is the only species documented to produce these pyrazines (Chuman *et al*., 1987; Robledo *et al*., 2014). The 2‐methyl‐6‐vinylpyrazine (41) was detected in the rectum of the fly (Chuman *et al*., 1987), while both pyrazines were detected in the air surrounding the fly (Robledo *et al*., 2014) and are thought to function as sex pheromones. *Anastrepha fraterculus* produces four different pyrazines: 2,5‐dimethyl‐pyrazine (3), 2,5‐dimethyl‐3‐methylpyrazine (4), 2,5‐dimethyl‐3‐ethylpyrazine (5), and 2,5‐dimethyl‐3‐*n*‐butylpyrazine (14) from the salivary gland (Lima, House & do Nascimento, 2001); this is the only study that detected pyrazines from the dipteran head. Robacker *et al*. (2009) identified 2,5‐dimethylpyrazine (3), 2,5‐dimethyl‐3‐methylpyrazine (4), and a novel compound 3,6‐dihydro‐2,5‐dimethylpyrazine (25), in the rectum of the sapote fruit fly (*A. serpentina*). This dihydro‐dimethyl pyrazine compound contains two double bonds instead of three in the pyrazine ring and is unique to *A. serpentina* within insects.

**Table 2:** Proposed functions of pyrazines and species in which they have been investigated. See Table S1 for chemical names of numbered compounds.

Order species	Pyrazine	Allo/pheromone (target species)	Functions	Reference
Phasmatodea				
*Phyllium westwoodii*	12,16,17	Allomones^b^	Defence^b^	Dossey *et al*. (2009)
Orthoptera				
*Locusta migratoria manilensis*	3	Pheromone	Aggregation^c^	Shi *et al*. (2011)
*Vestria* sp.	1,3,4	Allomones^b^	Defence^b^	Nickle *et al*. (1996)
Hemiptera				
*Triatoma dimidiata*	73,74,75	Pheromones^b^ Allomones^b^	Alarm^b^ Defence^b^	May‐Concha *et al*. (2015)
*Cercopis vulnerata*	5	Allomone^b^	Defence^b^	Körner (2006)
Lepidoptera				
*Arctia plantaginis*	73,74	Allomones (*Cyanistes caeruleus*)	Defence^c^	Rojas *et al*. (2017); Ottocento *et al*. (2023)
Coleoptera				
*Adalia bipunctata*	72,73,74	Pheromones	Aggregation	Susset *et al*. (2013)
*Hippodamia convergens*	72,73,74	Pheromones	Aggregation^c^	Wheeler & Cardé (2013)
*Coccinela septempunctata*	72	Pheromone	Aggregation	Al Abassi *et al*. (1998)
*Labidostomis lusitanica*	72	Pheromone	Aggregation^c^	López *et al*. (2023)
*Photuris trivittata*	73	Allomone (*Azteca lacrymosa*)	Defence^c^	Vencl *et al*. (2016)
*Lycus fernandezi*	72	Allomone (*Hylocichla* sp.)	Defence^ab^	Eisner *et al*. (2008)
*Lycus* sp.	72	Allomone (*Lycosa ceratiola*)	Defence^ab^	Eisner *et al*. (2008)
Diptera				
*Anastrepha curvicauda*	41,55	Pheromones	Sex^c^	Chuman *et al*. (1987); Robledo *et al*. (2014)
*Anastrepha fraterculus*	3,4,5,14	Pheromones^a^	Sex^a^	Lima *et al*. (2001)
*Bactrocera dorsalis*	22,23	Pheromones	Sex^c^	Ren *et al*. (2021)
*Bactrocera zonata*	4,5,22,23	Pheromones	Attractant^c^	Levi‐Zada *et al*. (2020)
Hymenoptera				
*Zaspilothynnus nigripes*	65	Pheromones	Sex	Bohman *et al*. (2012)
*Solenopsis invicta*	6	Pheromone	Sex/aggregation	Choi & Vander Meer (2015)
*Solenopsis invicta*	5,6	Pheromones	Alarm^c^	Vander Meer *et al*. (2010); Guan *et al*. (2014); Choi & Vander Meer (2015); Sun *et al*. (2017); Li *et al*. (2019)
*Calomyrmex* sp.	17	Pheromone	Alarm	Brown & Moore (1979)
*Hypoponera opacior*	17	Pheromone	Alarm	Duffield *et al*. (1976)
*Ponera pennsylvanica*	17	Pheromone	Alarm	Duffield *et al*. (1976)
*Wasmannia auropunctata*	16,36	Pheromones	Alarm	Showalter *et al*. (2010); Yu *et al*. (2014)
*Odontomachus troglodytes*	5,20,21	Pheromones	Alarm	Longhurst *et al*. (1978)
*Atta cephalotes*	5	Pheromone	Trail	Evershed & Morgan (1983)
*Atta sexdens rubropilosa*	5	Pheromone	Trail^c^	Cross *et al*. (1979); Evershed & Morgan (1983)
*Atta sexdens sexdens*	3,4,5	Pheromones	Trail^c^	Evershed & Morgan (1983); Billen *et al*. (1992); Morgan *et al*. (2013)
*Pogonomyrmex barbatus*	5	Pheromone	Trail	Hölldobler *et al*. (2001)
*Pogonomyrmex macricopa*	5	Pheromone	Trail	Hölldobler *et al*. (2001)
*Pogonomyrmex occidentalis*	5	Pheromone	Trail	Hölldobler *et al*. (2001)
*Pogonomyrmex rugosus*	5	Pheromone	Trail	Hölldobler *et al*. (2001)
*Manica rubida*	5	Pheromone	Trail^c^	Attygalle *et al*. (1986)
*Messor pergandei*	3,4	Pheromones	Trail	Plowes *et al*. (2014)
*Messor bouvieri*	5	Pheromone	Trail^c^	Jackson *et al*. (1989)
*Messor lobicornis*	5	Pheromone	Trail^c^	Evershed *et al*. (1982)
*Messor rubra*	5	Pheromone	Trail^c^	Evershed *et al*. (1982)
*Messor ruginodis*	5	Pheromone	Trail^c^	Evershed *et al*. (1982)
*Messor rugulosa*	5	Pheromone	Trail^c^	Evershed *et al*. (1982)
*Messor sabuleti*	5	Pheromone	Trail^c^	Evershed *et al*. (1982)
*Messor scabrinodis*	5	Pheromone	Trail^c^	Evershed *et al*. (1982)
*Messor schencki*	5	Pheromone	Trail^c^	Evershed *et al*. (1982)
*Messor sulcinodis*	5	Pheromone	Trail^c^	Evershed *et al*. (1982)
*Messor* sp.	5	Pheromone	Trail^c^	Jackson *et al*. (1991)
*Pheidole pallidula*	5	Pheromone	Trail^c^	Ali *et al*. (1988)
*Tetramorium caespitum*	3,5	Pheromones	Trail^c^	Attygalle & Morgan, (1983)
*Tetramorium meridionale*	4	Pheromone	Trail	Jackson *et al*. (1990)
*Eutetramorium mocquerysi*	9	Pheromone	Trail	Tentschert *et al*. (2000)

Trail functions include ‘recruitment’ in the cited study.

^a^ Results obtained from extract containing other chemical compounds.

^b^ Function derived from response to the release of the specific pyrazines.

^c^ Function was concentration dependent.

All *Bactrocera* species investigated (*B. cucumis*, *B. cucurbitae*, *B. dorsalis*, and *B. zonata*) also released 2,5‐dimethyl‐3‐methylpyrazine (4), and an additional pyrazine compound 2,5‐dimethyl‐3,6‐dimethylpyrazine (23) (Baker, Herbert & Lomer, 1982; Kitching *et al*., 1989; Levi‐Zada *et al*., 2020; Ren *et al*., 2021) was detected from the rectum. Subsequent research revealed that both pyrazines are synthesised by rectal microbes in *B. dorsalis*, which utilise glucose and threonine as substrates for their production (Ren *et al*., 2021). It is hypothesised that other *Bactrocera* species may utilise a similar pathway to produce these pyrazines. Most other *Bactrocera* species investigated also produce 2‐ethyl‐3,5,6‐trimethylpyrazine (22) from the rectum, and 2,5‐dimethyl‐3‐ethylpyrazine (5) has also been detected in the rectum of *B. cucurbitae*, *B. occipitalis*, and *B. zonata* (Perkins *et al*., 1990; Hadapad *et al*., 2016; Levi‐Zada *et al*., 2020). *B. cucurbitae* has been observed to produce the simple pyrazine methylpyrazine (1) in addition to other pyrazines detected in the rectum (Baker *et al*., 1982). This compound has only been detected in two other insects, both ants: European fire ant (*Manica rubida*) and *Tetramorium impurum* (Attygalle *et al*., 1986). One pyrazine was absent in *Anastrepha* but present in the rectum of *Bactrocera* and the abdomen of *Ceratitis capitata*: 2,6‐dimethyl‐3‐ethylpyrazine (6).

### (5) Lepidoptera

The occurrence of pyrazines in Lepidoptera is characterised by both a narrow taxonomic distribution, with records from only four families, and limitation to a few specific methoxypyrazines: 2‐methoxy‐3‐isopropylpyrazine (72), 2‐methoxy‐3‐*sec*‐butylpyrazine (73), and 2‐methoxy‐3‐isobutylpyrazine (74) (Table 1). In Erebidae and Zygaenidae moths, only one or two of these three pyrazines are found. In a species of *Amata* (Erebidae), 2‐methoxy‐3‐isopropylpyrazine (72) and 2‐methoxy‐3‐*sec*‐butylpyrazine (73) were reported (Rothschild, Moore & Brown, 1984; Moore *et al*., 1990). By contrast, other moths in Erebidae and Zygaenidae release 2‐methoxy‐3‐*sec*‐butylpyrazine (73) and 2‐methoxy‐3‐isobutylpyrazine (74). In Lymantriinae species, when provoked, larvae of *Lymantria dispar* have been observed to produce 2‐methoxy‐3‐isobutylpyrazine (74), and *Leucoma salicis* produces 2‐methoxy‐3‐isopropylpyrazine (72) (Aldrich *et al*., 1997). Some species, e.g. the cinnabar moth (*Tyria jacobaeae*, Erebidae) and a species of *Pollanisus* (Zygaenidae), have been found to produce only 2‐methoxy‐3‐*sec*‐butylpyrazine (73). The presence of the latter, and 2‐methoxy‐3‐isobutylpyrazine (74) were detected in whole‐body samples from *Arctia caja*, *Euplagia quadripunctaria*, and *Zygaena lonicerae* (Rothschild *et al*., 1984; Moore *et al*., 1990). Conversely, for *Arctia plantaginis*, methoxypyrazines have only been detected from reflex‐bleed fluid released from the prothorax region when provoked (Rojas *et al*., 2017; Burdfield‐Steel *et al*., 2018, 2020; Ottocento *et al*., 2023). This release of haemolymph is a defensive strategy against avian predators with pyrazines acting as an odour deterrent (Rojas *et al*., 2017; Ottocento *et al*., 2023). The pattern of occurrence of the three methoxypyrazines within Lepidoptera can not be explained by phylogenetic relatedness.

Pyrazines detected in Lepidoptera are mostly from adults, with a few only present in earlier life stages (larvae and/or pupae) (Table S2). Both sexes of *Heliconius charithonia* pupae produce all three methoxypyrazines, while in larvae, only 2‐methoxy‐3‐*sec*‐butylpyrazine (73) has been detected. Conversely, larvae of *Dryas iulia* had all three methoxypyrazines, while in pupae of *Papilio rumanzovia*, 2‐methoxy‐3‐*sec*‐butylpyrazine (73) and 2‐methoxy‐3‐isobutylpyrazine (74) were present (Moore *et al*., 1990). Some species release the same methoxypyrazines throughout their life stages. For example, in *Danaus plexippus* and *Heliconius melpomene* all three methoxypyrazines are found from larvae to adults. These compounds are detected in the claspers of males, and are transferred to mated females during copulation (Moore *et al*., 1990; Schulz *et al*., 2008).

### (6) Coleoptera

As is the case for Lepidoptera, pyrazines in Coleoptera are limited to a few methoxypyrazines, although they show a wider taxonomic distribution (Table 1). The methoxypyrazines most commonly detected in Coleoptera are: 2‐methoxy‐3‐isopropylpyrazine (72), 2‐methoxy‐3‐*sec*‐butylpyrazine (73), and 2‐methoxy‐3‐isobutylpyrazine (74). Studies have identified the presence of all three methoxypyrazines in various species within Coleoptera, particularly within the families Coccinellidae and Elateroidea (Table 1) (Moore *et al*., 1990; Al Abassi *et al*., 1998; Cudjoe, Wiederkehr & Brindle, 2005; Cai *et al*., 2007; Kögel *et al*., 2012; Susset *et al*., 2013; Wheeler & Cardé, 2013; Vencl *et al*., 2016; Schmidtberg *et al*., 2019). All three methoxypyrazines have been found in every life stage of *H. axyridis*, from egg to adult, implying that these compounds may be synthesised by microbes within the organism (Schmidtberg *et al*., 2019). Adult *H. axyridis* have been shown also to contain 2,5‐dimethyl‐3‐methoxypyrazine (75) (Cai *et al*., 2007), known only in one other insect: the hemipteran *Triatoma dimidiata* (May‐Concha *et al*., 2015).

Species with only a single methoxypyrazine are spread across various families (Table 1). For example, 2‐methoxy‐3‐isopropylpyrazine (72) has been identified from *Labidostomis lusitanica* (Chrysomellidae), *Rodatus boucardi* (Coccidulinae), *Epilachna curcurbidae* (Coccinellinae), and all Lycidae species investigated (Moore *et al*., 1990; Eisner *et al*., 2008; López *et al*., 2023). In the Lycidae, the only exception is *Metriorrhynchus rhipidius*, which has two other methoxypyrazines (Table 1). *M. rhipidius* exuded haemolymph containing three methoxypyrazines, 2‐methoxy‐3‐methylpyrazine (71), 2‐methoxy‐3‐isopropylpyrazine (72), and 2‐methoxy‐3‐*sec*‐butylpyrazine (73). The first of these has only been documented in this species among insects (Moore & Brown, 1981). It has been shown to act as a repellent against an avian predator, the red‐winged blackbird (*Agelaius phoeniceus*) (Avery & Nelms, 1990). In leaf beetles (*Labidostomis lusitanica*), 2‐methoxy‐3‐isopropylpyrazine (72) acts as an aggregation pheromone released by male beetles that attracts both males and females (López *et al*., 2023). Several species from multiple families are known to produce only 2‐methoxy‐3‐*sec*‐butylpyrazine (73) and *Coccinella transversalis* (Coccinellidae) and *Zonitis lutea* (Meloidae) have two methoxypyrazines (Table 1).

### (7) Hymenoptera

Hymenoptera has the highest number of species from which pyrazines have been recorded (Table 1). The majority are ants (Formicoidea), but they have also been recorded from bees (Apoidea) and wasps (Vespoidea, Tiphioidea and Thynnoidea) (Duffield *et al*., 1976; Evershed, Morgan & Cammaerts, 1982; Wheeler *et al*., 1982; Fales *et al*., 1984, 1988, 1992; Morgan *et al*., 1999; Hölldobler *et al*., 2001). The diversity and distribution of pyrazines across Hymenoptera differ among these higher taxonomic levels. Within species of Thynnoidea, Tiphioidea, Vespoidea, and Apoidea for which pyrazines have been recorded, approximately 80% (27/34 species) have 2,5‐dimethyl‐3‐isopentylpyrazine (17) (Table 1), which is known to function as an alarm pheromone (Table 2). The majority of pyrazines in bees and wasps have been detected from the head (i.e. mandibular glands) of examined individuals, with the exception of *Polybioides raphigastra* and *Parischnogaster mellyi* (Vespidae). For these species, pyrazines were detected in the venom glands (Dani *et al*., 1998; Sledge *et al*., 1999). Both species have been found to produce 2,5‐dimethylpyrazine (3) and 2,5‐dimethyl‐3‐methylpyrazine (4), which are commonly found in ants but not in other bees or wasps. Two alkyl‐pyrazines were found in *P. raphigastra* that are unique among Hymenoptera: 2‐ethyl‐3,5,6‐trimethylpyrazine (22) and 2,5‐dimethyl‐3,6‐dimethylpyrazine (23).

Several other pyrazines are found in bees and wasps in addition to 2,5‐dimethyl‐3‐isopentylpyrazine (17). In Apoidea, the European beewolf (*Philanthus triangulum*) produces 2,5‐dimethyl‐3‐*n*‐propylpyrazine (7) (Borg‐Karlson & Tengö, 1980), and *Epeolus* species release 2,5‐dimethyl‐3‐ethylpyrazine (5), 2,5‐dimethyl‐3‐isobutylpyrazine (12), and 2,5‐dimethyl‐3‐(2‐methylbutyl)‐pyrazine (16) from cephalic excretions (Tengö *et al*., 1982). Both 2,5‐dimethyl‐3‐*n*‐butylpyrazine (14) and 2,5‐dimethyl‐3‐isopentyl‐pyrazine (17) were found in mandibular glands of *Bicyrtes ventralis*, *Tachytes guatemalensis* (both Crabronidae), *Eremnophila aureonotata* (Sphecidae) and *Leptochilus acolhuus* (Vespidae) (Wheeler *et al*., 1982). Similarly, in the vespids *Ancistrocerus campestris*, *Euodynerus fuscus* and *Parancistrocerus* spp., 2,6‐dimethyl‐3‐*n*‐butylpyrazine (15) was found in the mandibular glands together with 2,5‐dimethyl‐3‐isopentylpyrazine (17). *Parancistrocerus fulvipes* produces two additional pyrazines, 2,5‐dimethyl‐3‐*n*‐propylpyrazine (7) and 2,6‐dimethyl‐3‐isopentylpyrazine (18) (Wheeler *et al*., 1982). *Ancistrocerus antilope* produces 2,3‐dimethyl‐5‐isobutylpyrazine (9) (Hefetz & Batra, 1980). The latter was found in head extracts of one other vespid species *Stenodynerus fulvipes*, which also appears to replace 2,6‐dimethyl‐3‐*n*‐butylpyrazine (15) with 2,5‐dimethyl‐3‐isobutylpyrazine (12) (Hefetz & Batra, 1980). One vespid wasp species, *Eumenes fraternus*, produces 2,5‐dimethyl‐3‐isopentylpyrazine (17) and 2,6‐dimethyl‐3‐isopentylpyrazine (18) (Hefetz & Batra, 1980; Wheeler *et al*., 1982). The thynnid wasp *Zaspilothynnus nigripes* produces a complex oxygenated pyrazine 2‐hydroxymethyl‐3‐(3‐methylbutyl)‐5‐methylpyrazine (65) together with 2,5‐dimethyl‐3‐isopentylpyrazine (17) (Bohman *et al*., 2014; Bohman & Peakall, 2014). Some species of bees and wasps do not produce 2,5‐dimethyl‐3‐isopentylpyrazine (17). *Ammophila urnaria* (Sphecidae) produces 2,6‐dimethyl‐3‐*n*‐propylpyrazine (8) and 2,6‐dimethyl‐3‐*n*‐butylpyrazine (15) (Duffield *et al*., 1981). The vespids *Pachodynerus erynnis* and *Stenodynerus floridanus* both produce two similar compounds with different orientations of 2,5 and 2,6 methyl groups: 2,5‐dimethyl‐3‐*n*‐propylpyrazine (7) and 2,6‐dimethyl‐3‐*n*‐propylpyrazine (8) (Wheeler *et al*., 1982).

Within Formicoidea, there are two distinct patterns of pyrazine composition: species with a very broad diversity of pyrazines, primarily within Ponerinae and occasionally in Ectatomminae; and those with a limited number of pyrazines, mainly from Myrmicinae, Formicinae, and Dolichoderinae (Table 1). In Myrmicinae, pyrazines have been recorded in 36 species, mostly from either the venom gland or mandibular glands. Almost all Myrmicinae species produce 2,5‐dimethyl‐3‐ethylpyrazine (5). In the genus *Myrmica*, all species have this pyrazine in the venom glands (Evershed *et al*., 1982; Jackson *et al*., 1991) except for *Myrmecia gulosa* which instead has 2,5‐dimethyl‐3‐*n*‐propyl‐pyrazine (7), found in the head (Brophy & Nelson, 1985). Genera such as *Manica*, *Messor*, *Daceton*, *Pogonomyrmex* and *Atta* additionally have 2,5‐dimethylpyrazine (3) and 2,5‐dimethyl‐3‐methylpyrazine (4) in the venom glands (Cross *et al*., 1979; Evershed & Morgan, 1983; Attygalle *et al*., 1986; Jackson, Wright & Morgan, 1989; Jackson *et al*., 1990; Mosquera & Oliveira, 1990; Billen, Beeckman & Morgan, 1992; Morgan *et al*., 1992, 2013; Hölldobler *et al*., 2001; Cruz‐López *et al*., 2006; Torres‐Contreras, Olivares‐Donoso & Niemeyer, 2007; Plowes *et al*., 2014; Silva‐Junior *et al*., 2018). A few species like *Manica rubida* and *Tetramorium impurum* produce a simple pyrazine, methylpyrazine (1), in addition to the three other pyrazines (Attygalle *et al*., 1986). *Solenopsis invicta* and *Acromyrmex octospinosus* have one pyrazine, 2,6‐dimethyl‐3‐ethylpyrazine (6) in addition to the ubiquitous 2,5‐dimethyl‐3‐ethylpyrazine (5) (Cross *et al*., 1982; Vander Meer, Preston & Choi, 2010; Guan *et al*., 2014; Sun *et al*., 2017; Hu *et al*., 2018; Li, Liu & Chen, 2019). These pyrazines are found in the venom gland in *A. octospinosus* (Cross *et al*., 1982) and in the mandibular glands in *S. invicta* (Vander Meer *et al*., 2010; Guan *et al*., 2014; Choi & Vander Meer, 2015; Sun *et al*., 2017; Hu *et al*., 2018; Li *et al*., 2019). A few species of Myrmicinae do not possess 2,5‐dimethyl‐3‐ethylpyrazine (5). In *Wasmannia auropunctata*, 2,5‐dimethyl‐3‐(2‐methylbutyl)‐pyrazine (16) and 3‐methyl‐2‐(2‐methylbutyl)‐pyrazine (36) were detected in head extracts (Showalter *et al*., 2010; Yu, Jang & Siderhurst, 2014). Other species that did not follow the general pattern include: *Eutetramorium mocquerysi*, which had 2,3‐dimethyl‐5‐isobutylpyrazine (9), 2,3‐dimethyl‐5‐(2‐methylbutyl)‐pyrazine (37), and 2,3‐dimethyl‐5‐(3‐methylbutyl)‐pyrazine (38) in the venom gland (Tentschert *et al*., 2000); *Aphaenogaster rudis* where the mandibular gland contained 5‐methyl‐3‐*n*‐propyl‐2‐(E‐1‐butenyl)‐pyrazine (45) and 5‐methyl‐3‐*n*‐propyl‐2‐(Z‐1‐butenyl)‐pyrazine (46) (Wheeler *et al*., 1982); and *Pheidole sinaitica* where the Dufour gland contained 3‐ethyl‐2,5‐dimethyl‐6‐propylpyrazine (26), 3‐ethyl‐2,5‐dimethyl‐6‐isopropylpyrazine (27), and 3‐ethyl‐2,5‐dimethyl‐6‐butylpyrazine (28) (Ali *et al*., 1988).

The chemical composition of pyrazines in the head of ants belonging to Formicinae, Ectatomminae, and Dolichoderinae frequently includes 2,5‐dimethyl‐3‐isopentylpyrazine (17), in common with Apidae (Table 1) (Brown & Moore, 1979; Brophy, Cavill & Duke, 1983; Brophy, 1989). Some species in the genera *Ectatomma*, *Rhytidoponera*, and *Calomyrmex* share common pyrazine combinations of 2,5‐dimethyl‐3‐isobutylpyrazine (12), 2,5‐dimethyl‐3‐(2‐methylbutyl)‐pyrazine (16), and 2,5‐dimethyl‐3‐isopentylpyrazine (17) (Brown & Moore, 1979; Brophy, 1989; Morgan *et al*., 1999). Several species in these subfamilies have specialised pyrazines with no common patterns between species. Species specificity and variability are discussed in Section V. One exceptional species, *Rhytidoponera metallica*, has at least 11 different pyrazines (Table 1), including 2,5‐dimethyl‐3‐(2‐methylbutyl)‐pyrazine (16), 2,5‐dimethyl‐3‐isopentylpyrazine (17), and 2,5‐dimethyl‐3‐citronellylpyrazine (44), and eight different alkenyl‐pyrazines (Brophy, Cavill & Plant, 1981; Tecle *et al*., 1987) that have not been documented in other insect species.

As previously mentioned, species within Ponerinae are exceptionally variable in pyrazines (Table 1), which all have been found in the head (mandibular glands). Species from the genera *Austroponera*, *Dinoponera*, *Pachycondyla*, and *Odontomachus* synthesise a particularly large number of different pyrazines, exceeding dozens in some cases. Such variation can be assumed to relate to complexity in chemical communications in highly interactive social insects. However, some species within Ponerinae are limited to one or a few pyrazines (Table 1). Such dramatic variation in the diversity of pyrazines among related species suggests that these pyrazines are likely to have very specific functions.

### (8) Other arthropods

Species outside of the class Insecta within the subphylum Hexapoda also possess complex pyrazine compounds. For example, within Collembola, dead individuals of *Onychiurus scotarius* and *O. circulans* contained 2,3‐dimethoxy‐pyrido[2,3‐*b*]‐pyrazine volatiles (Nilsson & Bengtsson, 2004); *Tetrodontophora bielanensis* release the same pyrazine and two others (3‐isopropyl‐2‐methoxypyrido[2,3‐b]‐pyrazine, and 2‐methoxy‐4H‐pyrido[2,3‐b]‐pyrazine‐3‐one) that serve as defensive chemicals (Dettner *et al*., 1996). Outside Hexapoda, some species within the subphylum Chelicerata also have pyrazines: the juvenile exocrine excretion of the harvestman *Holoscotolemon* sp. contains alkyl‐pyrazines, dimethyl‐isobutylpyrazines and a dimethyl‐isopentylpyrazine (Raspotnig *et al*., 2011).

## IV. FUNCTIONS OF PYRAZINES IN INSECTS

The functions of pyrazines detected in insects have been investigated through a variety of methods that include exposure of the pyrazines to potential intra‐ or interspecific receivers, including potential predators (Ottocento *et al*., 2023). The investigated pyrazines are either synthesised *in vitro* (Ottocento *et al*., 2023) or were sourced from extracts of glands or excretions (Burdfield‐Steel *et al*., 2020). In some cases, the function of pyrazines was assessed based on the emitter's behaviour, for example, when pyrazines were detected following a deliberate disturbance (Dossey *et al*., 2009), suggesting a potential link with defensive behaviour. Given this complexity, it is unsurprising that a wide range of functions have been suggested (see Table 2). Some pyrazines were proposed to serve a single function, whereas others demonstrated multifunctionality. While the existing body of research is limited (see Table 2), some generalisations have emerged.

### (1) Allomones

**Fig. 4:**
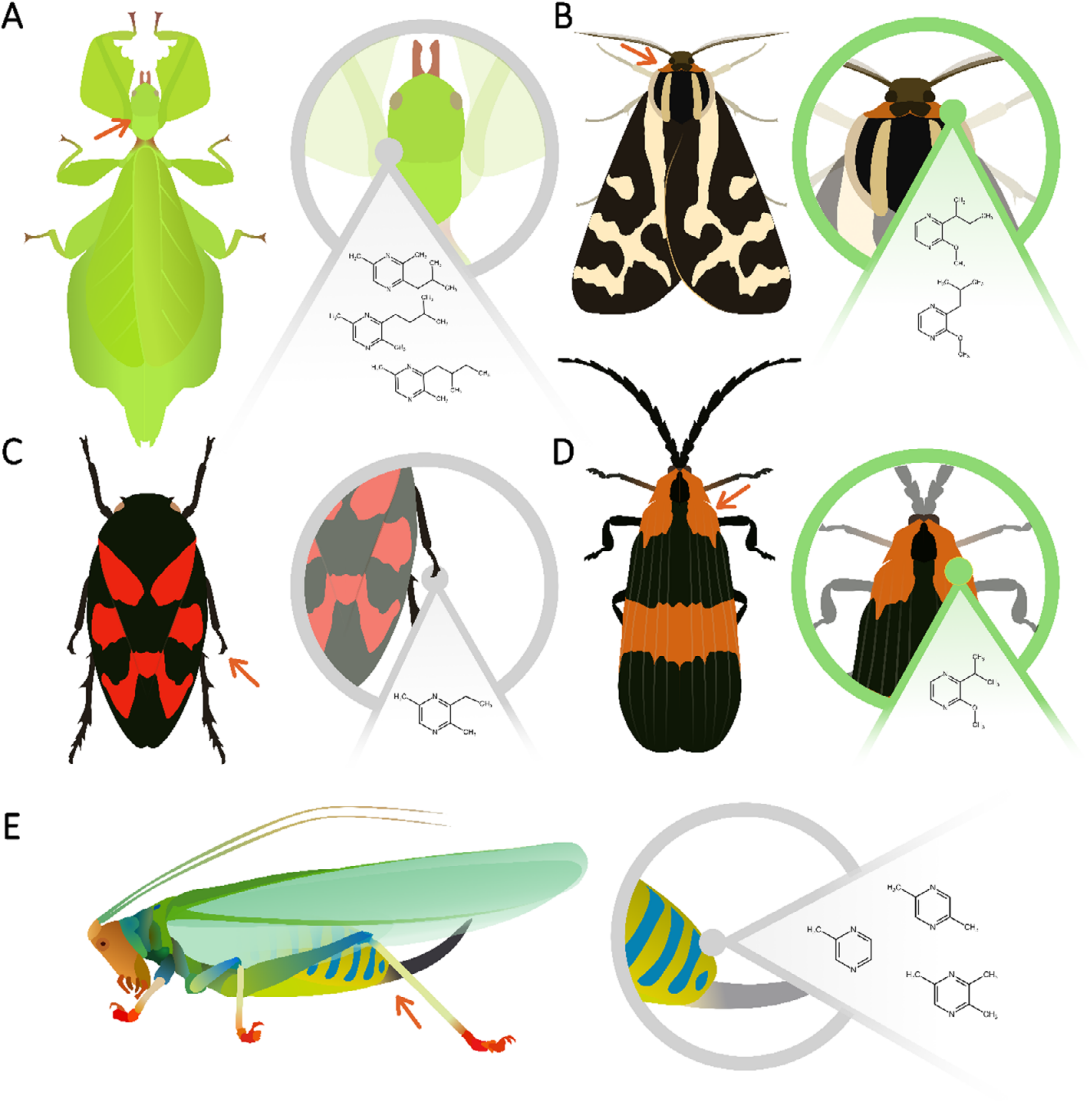
Example of pyrazine release in response to disturbance. The arrow indicates the site of the release, and the enlargement on the left indicates the pyrazine compounds released from it. (A) *Phyllium westwoodii* (Phasmatodea) releases a spray from glands in the prothorax that contains 2,5‐dimethyl‐3‐isobutylpyrazine (12), 2,5‐dimethyl‐3‐(2‐methylbutyl)‐pyrazine (16) and 2,5‐dimethyl‐3‐isopentylpyrazine (17). (B) *Arctia plantaginis* (Lepidoptera) reflex bleeds from the prothorax contain 2‐methoxy‐3‐*sec*‐butylpyrazine (73) and 2‐methoxy‐3‐isobutylpyrazine (74). (C) *Cercopis vulnerata* (Hemiptera) releases fluid containing 2,5‐dimethyl‐3‐ethylpyrazine (5) from between the tarsi. (D) *Calopteron reticulatum* (Coleoptera) reflex bleeds from the prothorax contain 2‐methoxy‐3‐isopropylpyrazine (72). (E) *Vestria* sp. (Orthoptera) odoriferous gland releases methyl‐pyrazine (1), 2,5‐dimethylpyrazine (3), and 2,5‐dimethyl‐3‐methylpyrazine (4).

Pyrazines that serve the explicit function of affecting the behaviour of other species (allomones), are found in all orders of insects except Diptera and Hymenoptera (Table 2). These pyrazines were either alkyl‐ or methoxy‐ substituted pyrazines. These pyrazines are emitted from the body in response to a threat, as a spray [e.g. *Phyllium westwoodii* (Phasmatodea)] (Dossey *et al*., 2009) (Fig. 4A) or in droplets from various body parts depending on the species (Rothschild, 1961; Nickle *et al*., 1996; Körner, 2006; Burdfield‐Steel *et al*., 2018) (Fig. 4B–E). This behaviour suggests that the pyrazines act as allomones, in defence against predators. The advantages of using pyrazines in this way are their high volatility, enabling them to reach a predator before attack or consumption. Hence, they function as a warning odour, targeting the olfactory system or are repellent to taste (Dossey *et al*., 2009; Rojas *et al*., 2017).

Methoxypyrazines are the main pyrazines that function as allomones in Lepidoptera and Coleoptera (Table 2). Studies that have explored the role of methoxypyrazines against potential predators have shown that they can act as target‐specific agents in an anti‐predation context. Two methoxypyrazines, 2‐methoxy‐3‐*sec*‐butylpyrazine (73) and 2‐methoxy‐3‐isobutylpyrazine (74), found in reflex fluid from the wood tiger moth (*Arctia plantaginis*; Lepidoptera) repel avian predators but not predatory invertebrates, including *Trichonephila senegalensis* (Araneidae; Araneae) and *Formica* sp. (Formicidae; Hymenoptera) (Rojas *et al*., 2017; Burdfield‐Steel *et al*., 2020). Interestingly, some studies support the opposite effect: the tropical firefly *Photuris trivittata* (Coleoptera) secretes 2‐methoxy‐3‐*sec*‐butylpyrazine (73), which repels neotropical *Azteca* ants (Formicidae; Hymenoptera) (Vencl *et al*., 2016). Methoxypyrazines have also been shown to act as repellents against bats (*Myotis nigricans* and *Molossus molossus*; Chiroptera) but not against cane toads (*Rhinella marina*; Amphibia) (Vencl *et al*., 2016).

The effectiveness of methoxypyrazines as repellents can be concentration dependent. Increased 2‐methoxy‐3‐*sec*‐butylpyrazine (73) emitted by *P. trivittata* and *A. plantaginis* repelled avian predators (Vencl *et al*., 2016; Ottocento *et al*., 2023), while in the latter higher concentrations of 2‐methoxy‐3‐isobutylpyrazine (74) reduced repellence against birds (Ottocento *et al*., 2023) (Table 2). Thus, the functions of pyrazines are not necessarily directly related to their concentration (Ottocento *et al*., 2023). Combination with other chemicals, including sequestered pyrrolizidine alkaloids, can enhance their effects (Winters *et al*., 2021). Overall, the use of pyrazines as allomones is typically limited to defence against predation and is found in a limited number of insect species.

### (2) Pheromones

**Fig. 5:**
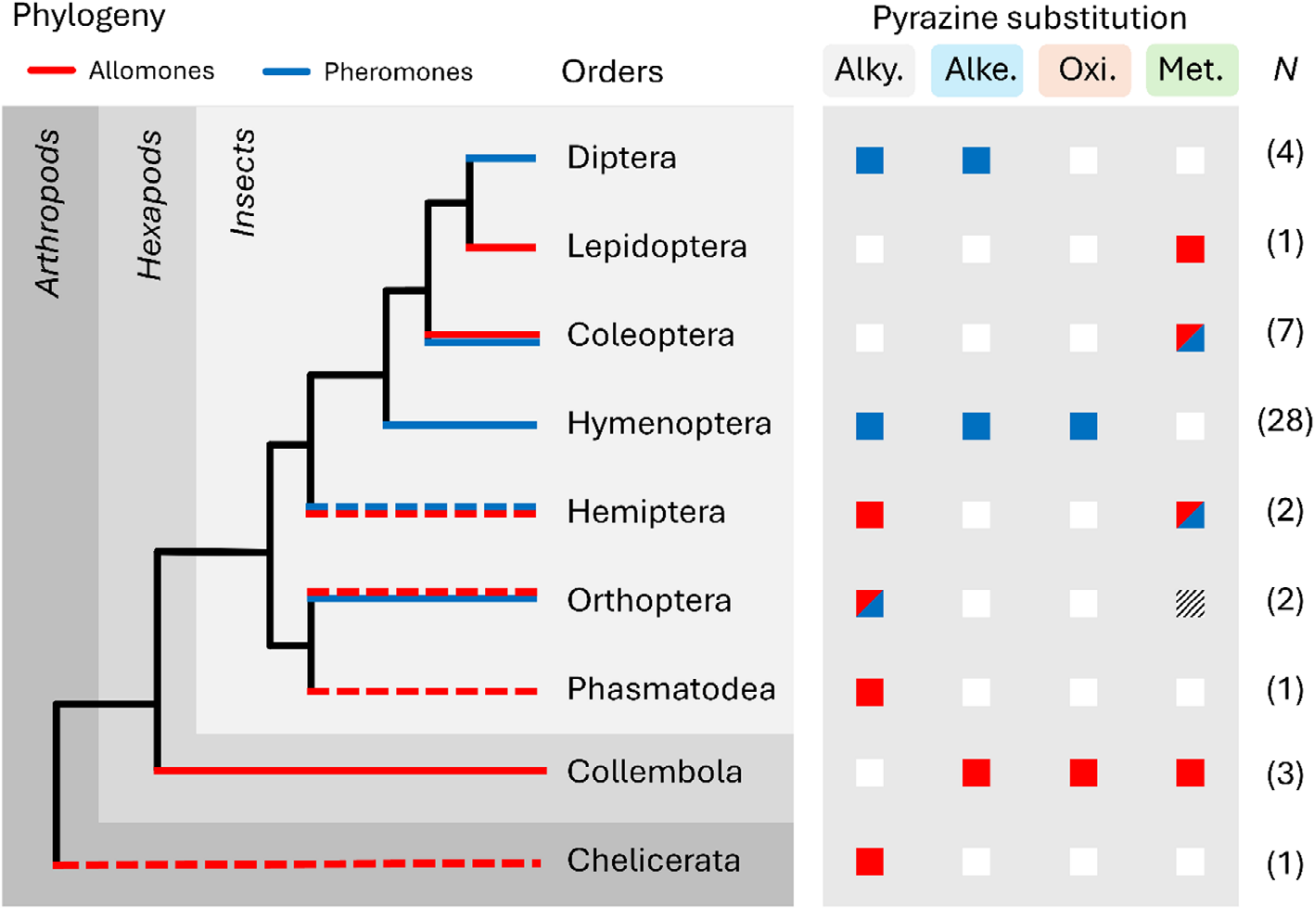
The occurrence of pyrazines and associated functions as allomones (red) and pheromones (blue) in Arthropoda. Phylogeny based on Misof *et al*. (2014). Dashed lines indicate that the hypothesised functions have not been examined. The pyrazines occurring in the group of insects are categorised by numbers and substitution groups by letters (Alky. = alkyl‐pyrazine; Alke. = alkenyl‐pyrazine; Oxi. = oxygenated pyrazine, Met. = methoxypyrazine), and coloured by the group of functions (note both functions are found in some taxa for some pyrazines). Pyrazine categories with unknown functions are represented by the diagonally hatched square. The number of species in which the functions have been shown is indicated in parentheses on the right (*N*). See Table 2 for further details.

It is well known that pyrazines can alter the behaviour of conspecifics (i.e. act as pheromones) (Table 2). Pyrazines functioning as pheromones have substituents from all groups in a pattern that is highly specific to each insect order (Fig. 5). Pheromones in Coleoptera and Hemiptera are all methoxypyrazines that function as alarm and aggregation pheromones, while other orders, like Diptera, use alkyl‐ and alkenyl‐pyrazines as sex/attractant pheromones. Hymenoptera and Orthoptera produce alkyl‐pyrazines with a variety of functions as pheromones (Table 2).

Methoxypyrazines produced in Coleoptera and Hemiptera have opposite functions. In *Triatoma dimidiata* (Hemiptera), methoxypyrazines function to alarm conspecifics and induce dispersal (May‐Concha *et al*., 2015), while in *Labidostomis lusitanica* (Coleoptera; Chrysomellidae) and *Adalia bipunctata* (Coccinellidae) the same methoxypyrazines are used as signals to aggregate (Susset *et al*., 2013; López *et al*., 2023). Interestingly, a third methoxypyrazine is present that differs between the hemipteran and coleopteran species (Table 2), and these combinations also function as allomones.

Alkyl‐pyrazines in Hymenoptera function broadly as pheromones, for sex, alarm, trail following and aggregation (Table 2). However, only a limited number of alkyl‐pyrazines appear to be multifunctional. For example, 2,5‐dimethyl‐3‐ethylpyrazine (5) functions as an alarm and trail pheromone in numerous species, whereas 2,6‐dimethyl‐3‐ethylpyrazine (6) functions as both a sex/aggregation and alarm pheromone in the fire ant (*Solenopsis invicta*) (Choi & Vander Meer, 2015). Interestingly, the same pheromones are also used by a parasitoid phorid fly (*Pseudacteon tricuspis*) to locate the red fire ant nest (Sharma, Vander Meer & Fadamiro, 2011). The majority of pheromone functions are recognised in the ants (Formicoidea). Each subfamily has its own specific combination of pyrazines used as alarm pheromones, whereas those used as trail pheromones are broadly similar across subfamilies. Studies have also reported that some ant species will follow the trails of other species, supporting this lack of diversification of pyrazines as trail pheromones (Cross *et al*., 1982). Outside Formicoidea, the oxygenated pyrazine 2‐hydroxymethyl‐3‐(3‐methylbutyl)‐5‐methylpyrazine (65) is used as a sex pheromone by *Zaspilothynnus nigripes* (Thynnoidea) (Bohman *et al*., 2012). This pheromone is noteworthy for its use in a sexual deception: it is emitted by an orchid (*Drakaea linvida*) to attract males to pollinate its flowers (Bohman *et al*., 2014).

Among Diptera, pyrazines function only as sex pheromones (Table 2). All the species with known functions of pyrazines have unique pyrazine combinations that are essential components of pheromone blends (Lima *et al*., 2001). For Orthoptera, there are only limited reports on the role of pyrazines as pheromones. However, 2,5‐dimethylpyrazine (3) is known to elicit aggregation of the oriental migratory locust (Shi *et al*., 2011). This pyrazine is released from faecal samples from the locust and is not directly released to facilitate aggregation behaviour.

Following the recognition of pyrazines as pheromones, researchers conducted experiments testing different concentrations. The results indicate a positive response to increasing concentrations of aggregation, alarm, and sex pheromones (Lima *et al*., 2001; Wheeler & Cardé, 2013; Robledo *et al*., 2014; Levi‐Zada *et al*., 2020; López *et al*., 2023). By contrast, mixed results were found for pyrazines used as trail pheromones. In the genera *Messor* and *Pheidole*, there was a positive response to the trail pheromones with increasing concentration (Evershed *et al*., 1982; Ali *et al*., 1988). By contrast, for species of *Atta* and *Tetramorium*, the response took the form of a bell‐shaped curve, with an optimal concentration and reduced effectiveness of pheromones at higher and lower concentrations (Attygalle & Morgan, 1983; Jackson *et al*., 1989; Morgan *et al*., 2013).

The functions of chemical communication *via* pheromones vary among insects, encompassing interactions between sexes, groups, and families (Leonhardt *et al*., 2016). As elaborated herein, pyrazines are clearly efficacious chemical communication instruments for insects. Leonhardt *et al*. (2016) provide a comprehensive overview of chemical communication within different insect orders, that we summarise in Table 3. Their study identified pyrazines as pheromones in a wide range of insect taxa and across all interaction scales, with notable functions including aggregation, trail following, and alarm. However, in Diptera and Hymenoptera, pyrazines function specifically as pheromones in sexual interactions. Among Hymenoptera, pheromones are exceptionally diverse, and it is within this group that the highest number of different pyrazines have been detected (Table 1). This pyrazine diversity may enhance their use as pheromones in social insects, perhaps driving the diversification of communication systems. However, note that the diversity of pyrazines and the number of species in which they are present may be underestimated due to the limited research conducted on other orders, particularly Phasmatodea and Orthoptera.

**Table 3:** Overview of the use of chemical communication across different orders of insects, and its association with pyrazine variations and species known to possess pyrazines. Grey highlighted cells indicate a known function of pyrazines as pheromones. Data are derived from Leonhardt *et al.* (2016). The range of chemical communication is categorised by the scale of the recipients: between sexes, within sexes, among groups, or among families. X = present; − = not known; ? = undetermined.

Orders	Number of pyrazines	Number of species	Range of chemical communication			
			Between sexes	Within sexes	Among groups	Among families
Diptera	11	9	X	X	X	–
Lepidoptera	3	20	X	X	X	–
Coleoptera	4	29	X	X	X	X
Hymenoptera	67	112	X	X	X	X
Hemiptera	5	11	X	X	X	–
Orthoptera	4	5	X	X	X	–
Phasmatodea	3	1	?	–	–	–

## V. VARIATION OF PYRAZINES: AN EXPLORATION OF ONTOGENY AND INTRA‐INDIVIDUAL VARIATION

The majority of studies examining semiochemicals in insects focus on adults, as chemical communication is widely regarded as being particularly significant during this stage. This is particularly evident for studies on pyrazines in insects, where 97% of the pyrazines identified were from adults (Table S2). However, only a limited number of studies have examined earlier life stages (Moore *et al*., 1990; Schmidtberg *et al*., 2019). A comprehensive evaluation of pyrazines in the different life stages of insects remains to be conducted, and the specific functions of pyrazines in adults still require investigation for many species. Among the few studies that have examined pyrazines in earlier life stages, Coleoptera and Lepidoptera are known to produce methoxypyrazines in larvae and/or eggs (Table S2). Methoxypyrazines in both orders are highly likely to function as allomones, particularly as warning signals to predators, which is as important for larvae as it is for adults.

Pyrazines are known to vary between the sexes and among adult castes in eusocial insects that have been studied. For example, in Diptera, the majority of pyrazines are found in males, as they function primarily as sex pheromones (Chuman *et al*., 1987). In Hymenoptera, workers produce significantly higher amounts of pyrazines than other castes. This phenomenon may reflect the intricate nature of the social interactions among workers engaged in tasks within colonies, such as trail following and the use of alarm pheromones (Hu *et al*., 2018). Further exploration of pyrazines in earlier life stages remains necessary to elucidate the underlying mechanisms that regulate their production and the potential functions they may serve.

The identification of the origin of pyrazines within the insect body also remains necessary for a comprehensive understanding of the functions of these compounds. The anatomical location of pyrazine production can serve as an indicator of its potential role. The preliminary screening of insects for pyrazines typically involves the extraction of these compounds from the whole body; this is the most common method used in studies of Lepidoptera and Coleoptera (Table S3). In other insects, pyrazines have been identified in different parts of the body including secretion glands, haemolymph, and the gut. Pyrazine screening in Diptera has focused primarily on the abdominal region, where the sex pheromones are likely to be produced. In species known to excrete fluid as allomones, the haemolymph or release sites have been investigated, such as the prothorax in Lepidoptera. In Hymenoptera, the detection of pyrazines in different body segments such as the head and gaster region reflects the functions of their pheromones. The head region, including the mandibular glands, of Hymenoptera is responsible for the production of alarm pheromones (e.g. pyrazines 16, 17, 20, 21, and 36 in Table 2), whereas trail pheromones are released from the gaster region, where the venom/poison and Dufour's glands are located (Table S3). The alkyl‐pyrazines 2,5‐dimethyl‐3‐ethylpyrazine (5) and 2,6‐dimethyl‐3‐ethylpyrazine (6), which function as both alarm and trail pheromones, have been detected in both regions.

The extraction of pyrazines from the whole body may make it difficult to determine whether the pyrazines are derived from the insect itself or are sequestered from their food. Many insects consume different food in their earlier life stages compared to the adult stage, raising the possibility of variation in the presence or absence of pyrazines across different life stages resulting from these dietary sources. Exploring the presence of pyrazines in the diet will be required to elucidate the potential sources of these pyrazines.

## VI. EVOLUTION OF PYRAZINES IN INSECTS

Exploring the evolution of semiochemicals is complex and often requires a detailed understanding of their synthesis and the associated genes (Rebholz *et al*., 2023). From Fig. 5, we can assess potential patterns of pyrazine evolution across function categories and pyrazine substitution groups. Hemimetabolous insects, such as Phasmatodea, Orthoptera, and Hemiptera, predominantly exhibit pyrazines with an alkyl group, with certain species within Hemiptera also known to have methoxypyrazines. By contrast, Hymenoptera appear to lack methoxypyrazines, suggesting an evolutionary trajectory in which alkyl‐pyrazines diversified into alkenyl‐ and oxygenated pyrazines. In Coleoptera and Lepidoptera, methoxypyrazines are present in allomones/pheromones, while they appear to be absent in Diptera. Alkyl‐ and alkenyl‐pyrazines as pheromones have undergone a similar diversification process in Diptera to that in Hymenoptera. The rather limited evidence on the phylogenetic distribution of species producing pyrazines has hindered the development of more detailed insights into the evolution of pyrazine production.

## VII. SYNTHESIS OF PYRAZINES IN INSECTS

Recent investigations of pyrazine biosynthesis in insects have led to the identification of novel pathways and associated candidate organisms involved in their synthesis, including microbes and symbionts (Table 4). Currently, pyrazines are thought to be biosynthesised through two mechanisms: *de novo* production by the organism or microbe‐associated production. Both pathways have been suggested for various insect orders (Table 4). A recent focus has been on understanding the microbiomes of insects through the study of endogenously synthesised pyrazines. It is noteworthy that species classified as performing *de novo* pyrazine synthesis may actually be utilising microbial synthesis, and there is clear potential for pyrazines to be produced without requiring dietary sources. Several species from Arctiinae and Zygaenidae have been reared on host plants that did not contain pyrazines, but were found to produce three different methoxypyrazines (Table 4) (Moore *et al*., 1990). *Arctia plantaginis*, when reared on an artificial diet that excluded all plant materials, still produced two methoxypyrazines: 2‐methoxy‐3‐*sec*‐butylpyrazine (73), and 2‐methoxy‐3‐isobutylpyrazine (74) (Burdfield‐Steel *et al*., 2018). An ability to produce allomones independently is clearly advantageous as it would remove a dietary dependence (Rojas *et al*., 2017), although synthesis presumably will require a significant energy investment. The same methoxypyrazines detected in Lepidoptera are known to be synthesised in the gut of *Harmonia axyridis* (Coleoptera) *via* symbiotic microbes (Schmidtberg *et al*., 2019). It is thus possible that symbionts in Lepidoptera also may facilitate the production of pyrazines, but this requires further investigation.

**Table 4:** Known biosynthesis of pyrazines across insects. See Table S1 for chemical names of numbered pyrazines.

Order/species	Pyrazine number	Synthesis method	Reference
Lepidoptera			
*Arctia plantaginis*	73,74	*De novo* ^a^	Burdfield‐Steel *et al*. (2018)
*Arctia caja*	73,74	*De novo* ^b^	Moore *et al*. (1990)
*Euplagia quadripunctaria*	73,74		
*Tyria jacobaeae*	73		
*Amata* sp.	72,73		
*Pollanisus* sp.	73		
*Zygaena lonicerae*	73,74		
Coleoptera			
*Harmonia axyridis*	72,73,74	Microbial	Schmidtberg *et al*. (2019)
Diptera			
*Bactrocera dorsalis*	4,23	Microbial	Ren *et al*. (2021)
Hymenoptera			
*Atta sexdens rubropilosa*	3,5	Microbial	Silva‐Junior *et al*. (2018)

^a^ Pyrazines detected in organisms reared on an artificial diet (without pyrazine or plant matter in the diet).

^b^ Pyrazines detected in organisms reared on plants that do not contain those specific pyrazines.

The synthesis of pyrazines by microbes has been observed in Coleoptera, Diptera and Hymenoptera to date (see Table 4). Symbiotic microbes present in the gut of *H. axyridis* (Coleoptera) produce 2‐methoxy‐3‐isopropylpyrazine (72), 2‐methoxy‐3‐*sec*‐butylpyrazine (73) and 2‐methoxy‐3‐isobutylpyrazine (74). The bacterial genera *Serratia* and *Lactococcus* have been identified as potential candidates for the production of these compounds (Schmidtberg *et al*., 2019). In Diptera, the synthesis of sex pheromones such as 2,5‐dimethyl‐3‐methylpyrazine (4) and 2,5‐dimethyl‐3,6‐dimethylpyrazine (23) occurs in the rectum of *Bactrocera dorsalis* fruit flies. It is likely that bacteria in the genus *Bacillus* produce these pyrazines in the presence of glucose and threonine (Ren *et al*., 2021). Another notable instance of microbial synthesis of pyrazines is found in the leaf‐cutter ant (*Atta sexdens rubropilosa*; Hymenoptera), which harbours associated bacteria that produce two pyrazines; 2,5‐dimethylpyrazine (3) and 2,5‐dimethyl‐3‐ethylpyrazine (5) (Silva‐Junior *et al*., 2018). These pyrazines serve as trail pheromones, and are released from the venom gland in the gaster region. By contrast, other species of ponerine ants are predatory and therefore do not consume plants that could provide a source of pyrazines. This suggests that the pyrazines in most ponerine ants are synthesised *de novo* or with the assistance of their microbiota (Fales *et al*., 1988).

## VIII. FUTURE DIRECTIONS IN THE STUDY OF PYRAZINES IN INSECTS

Understanding of the identification and synthesis of pyrazines present in insects has improved significantly since it was last reviewed (Brophy, 1989). Novel analytical instruments have facilitated the identification of previously unrecognised pyrazines (Cai *et al*., 2007; Mayorga‐Martino *et al*., 2025; Khashaveh *et al*., 2025). However, challenges regarding extraction of pyrazines persist, given their low concentrations in insects (0.1–100 ng) (Morgan *et al*., 1999). Indeed, to obtain sufficient quantities of pyrazines for analysis, hundreds of individuals are required. In some cases, previously detected pyrazines have been found to be misidentified and subsequently corrected (Showalter *et al*., 2010; Xu *et al*., 2018). Further exploration of pyrazines in insects could lead to the discovery of new pyrazines and provide a more accurate description of those present in each species.

## IX. CONCLUSIONS

(1) Pyrazines have been identified to date in seven insect orders. Hemimetabolous insects, such as Phasmatodea, Orthoptera, and Hemiptera typically produce alkyl‐substituted pyrazines, with some Hemiptera and Orthoptera also producing methoxy‐ variants. Methoxy‐substituted pyrazines are absent in Hymenoptera but present in Coleoptera and Lepidoptera, where they serve as both pheromones and allomones. In Diptera, pyrazines are only known from a few species, and have alkyl or alkenyl substituents. Pheromonal pyrazines are predominantly substituted with alkyl and alkenyl groups, whereas allomonal pyrazines more often feature methoxy groups.
(2) Social insects, particularly within the order Hymenoptera, exhibit a greater diversity and frequency of pyrazine use compared to solitary species and other insect orders. This diversity may be associated with the complexity of communication systems required by eusociality.
(3) Current evidence suggests that pyrazines are synthesised in insects, possibly by microbial symbionts, rather than arising from dietary intake or environmental acquisition. Pyrazines are mainly associated with adult stages, suggesting a predominant role in later‐life communication, although there is limited information for early life stages. Current knowledge regarding the biosynthesis of pyrazines is limited, and further research on the pathways involved is necessary for an understanding of the evolution of pyrazine synthesis.
(4) Further research is needed to clarify the distribution of pyrazines across insects, and the evolution and functions of pyrazines. Exploration of pyrazine distribution across a broader range of insect orders could provide valuable insights into the prevalence of these compounds. A thorough evaluation of their biological functions is needed to enable a comprehensive understanding of the roles of pyrazines in insect biology.

## Supporting information


**Table S1.** Pyrazine chemical name and the substitution groups of pyrazines detected within insects (see Table 1).
**Table S2.** Number of species in which pyrazines have been recorded across ontogeny stages.
**Table S3.** Anatomical sites in which the presence of pyrazines has been detected across insects.
**Fig. S1.** Chemical structures of pyrazine compounds detected within insects.

## Data Availability

Data sharing not applicable to this article as no datasets were generated or analysed during the current study.
