# Supplementary material for: Ecology and evolution of pyrazines in insects
Source: Biol Rev Camb Philos Soc. 2026 Mar 20;101(4):1937–59. doi: 10.1002/brv.70160 (PMC13326771; doi:10.1002/brv.70160)

**Supporting Information**

**Table S1.** Pyrazine chemical name and substitution groups of pyrazines detected within insects (see Table 1). See Fig. 1 for substitution category.

| **Substitution category** | **Pyrazine number** | **Compound name** |
| --- | --- | --- |
| Alkyl- | 1 | methylpyrazine |
| Alkyl- | 2 | 2,6-dimethylpyrazine |
| Alkyl- | 3 | 2,5-dimethylpyrazine |
| Alkyl- | 4 | 2,5-dimethyl-3-methylpyrazine |
| Alkyl- | 5 | 2,5-dimethyl-3-ethylpyrazine |
| Alkyl- | 6 | 2,6-dimethyl-3-ethylpyrazine |
| Alkyl- | 7 | 2,5-dimethyl-3-*n*-propylpyrazine |
| Alkyl- | 8 | 2,6-dimethyl-3-*n*-propylpyrazine |
| Alkyl- | 9 | 2,3-dimethyl-5-isobutylpyrazine |
| Alkyl- | 10 | 2,5-dimethyl-3-*sec*-butylpyrazine |
| Alkyl- | 11 | 2,6-dimethyl-3-*sec*-butylpyrazine |
| Alkyl- | 12 | 2,5-dimethyl-3-isobutylpyrazine |
| Alkyl- | 13 | 2,6-dimethyl-3-isobutylpyrazine |
| Alkyl- | 14 | 2,5-dimethyl-3-*n*-butylpyrazine |
| Alkyl- | 15 | 2,6-dimethyl-3-*n*-butylpyrazine |
| Alkyl- | 16 | 2,5-dimethyl-3-(2-methylbutyl)-pyrazine |
| Alkyl- | 17 | 2,5-dimethyl-3-isopentylpyrazine |
| Alkyl- | 18 | 2,6-dimethyl-3-isopentylpyrazine |
| Alkyl- | 19 | 2,5-dimethyl-3-*n*-pentylpyrazine |
| Alkyl- | 20 | 2,6-dimethyl-3-*n*-pentylpyrazine |
| Alkyl- | 21 | 2,6-dimethyl-3-*n*-hexylpyrazine |
| Alkyl- | 22 | 2-ethyl-3,5,6-trimethylpyrazine |
| Alkyl- | 23 | 2,5-dimethyl-3,6-dimethylpyrazine |
| Alkyl- | 24 | 2,5-dimethyl-3,6-diisopentylpyrazine |
| Alkyl- | 25 | 3,6-dihydro-2,5-dimethylpyrazine |
| Alkyl- | 26 | 3-ethyl-2,5-dimethyl-6-propylpyrazine |
| Alkyl- | 27 | 3-ethyl-2,5-dimethyl-6-isopropylpyrazine |
| Alkyl- | 28 | 3-ethyl-2,5-dimethyl-6-butylpyrazine |
| Alkyl- | 29 | 2,5-dimethyl-3-(2-methylhexyl)-pyrazine |
| Alkyl- | 30 | 2,5-dimethyl-3-*n*-hexylpyrazine |
| Alkyl- | 31 | 2,5-dimethyl-3-isohexylpyrazine |
| Alkyl- | 32 | 2,5-dimethyl-3-*n*-heptylpyrazine |
| Alkyl- | 33 | 2,5-dimethyl-3-isoheptylpyrazine |
| Alkyl- | 34 | 2,5-dimethyl-3-*n*-octylpyrazine |
| Alkyl- | 35 | 2,5-dimethyl-3-isooctylpyrazine |
| Alkyl- | 36 | 3-methyl-2-(2-methylbutyl)-pyrazine |
| Alkyl- | 37 | 2,3-dimethyl-5-(2-methylbutyl)-pyrazine |
| Alkyl- | 38 | 2,3-dimethyl-5-(3-methylbutyl)-pyrazine |
| Alkyl- | 39 | 2,6-dimethyl-3-*n*-heptylpyrazine |
| Alkyl- | 40 | 2,6-dimethyl-3-isohexylpyrazine |
| Alkenyl- | 41 | 2-methyl-6-vinylpyrazine |
| Alkenyl- | 42 | 2,5-dimethyl-E-3-styrylpyrazine |
| Alkenyl- | 43 | 2,5-dimethyl-Z-3-styrylpyrazine |
| Alkenyl- | 44 | 2,5-dimethyl-3-citronellylpyrazine |
| Alkenyl- | 45 | 5-methyl-3-*n*-propyl-2-(E-1-butenyl)-pyrazine |
| Alkenyl- | 46 | 5-methyl-3-*n*-propyl-2-(Z-1-butenyl)-pyrazine |
| Alkenyl- | 47 | 5-methyl-3-isopentyl-2-(E-3-methylpent-1-enyl)-pyrazine |
| Alkenyl- | 48 | 5-methyl-3-(2-methylbutyl)-2-(E-3-methylpent-1-enyl)-pyrazine |
| Alkenyl- | 49 | 5-methyl-3-isopentyl-2-(Z-3-methylpent-1-enyl)-pyrazine |
| Alkenyl- | 50 | 5-methyl-3-(2-methylbutyl)-2-(Z-3-methylpent-1-enyl)-pyrazine |
| Alkenyl- | 51 | 5-methyl-3-isopentyl-2(E-3-methylbuten-1-enyl)-pyrazine |
| Alkenyl- | 52 | 5-methyl-3-(2-methylbutyl)-2-(E-3-methylbut-1-enyl)-pyrazine |
| Alkenyl- | 53 | 5-methyl-3-(2-methylbutyl)-2-(Z-3-methylbut-1-enyl)-pyrazine |
| Alkenyl- | 54 | 5-methyl-3-isopentyl-2-(Z-3-methylbuten-1-enyl)-pyrazine |
| Alkenyl- | 55 | 2,5-dimethyl-3-vinylpyrazine |
| Alkenyl- | 56 | 2,5-dimethyl-3-isopentyl-6-(E-isopent-1-enyl)-pyrazine |
| Alkenyl- | 57 | 2,5-dimethyl-3-isopentyl-6-(Z-isopent-1-enyl)-pyrazine |
| Alkenyl- | 58 | 2,5-dimethyl-3-isopentyl-6-(isopent-2-enyl)-pyrazine |
| Oxygenated- | 59 | 2,5-dimethyl-Oxygenated-,6-di(1-hydroxyisopentyl)-pyrazine |
| Oxygenated- | 60 | 2,5-dimethyl-3-(1-oxoisopentyl)-6-(1-hydroxyisopentyl)-pyrazine |
| Oxygenated- | 61 | 2,5-dimethyl-3,6-di(1-oxoisopentyl)-pyrazine |
| Oxygenated- | 62 | 2,5-dimethyl-3-isopentyl-6-(1-hydroxyisopentyl)-pyrazine |
| Oxygenated- | 63 | 2,5-dimethyl-3-(1-oxoisopentyl)-6-(1-oxoisopent-2-enyl)-pyrazine |
| Oxygenated- | 64 | 2,5-dimethyl-3-(1-hydroxyisopentyl)-6-(1-oxoisopent-2-enyl)-pyrazine |
| Oxygenated- | 65 | 2-hydroxymethyl-3-(3-methylbutyl)-5-methylpyrazine |
| Oxygenated- | 66 | 2,5-dimethyl-3-(1-hydroxy-3-methylbutyl)-6-(2-methylpropyl)-pyrazine |
| Oxygenated- | 67 | 2,5-dimethyl-3-isopentyl-6-(1-hydroxyisobutyl)-pyrazine |
| Oxygenated- | 68 | 2,5-dimethyl-3-isopentyl-6-(1-hydroxybutyl)-pyrazine |
| Oxygenated- | 69 | 2,5-dimethyl-3-isopentyl-6-(1-hydroxypropyl)-pyrazine |
| Oxygenated- | 70 | 2,5-dimethyl-3-isobutyl-6-(1-hydroxypropyl)-pyrazine |
| Methoxy- | 71 | 2-methoxy-3-methylpyrazine |
| Methoxy- | 72 | 2-methoxy-3-isopropylpyrazine |
| Methoxy- | 73 | 2-methoxy-3-*sec*-butylpyrazine |
| Methoxy- | 74 | 2-methoxy-3-isobutylpyrazine |
| Methoxy- | 75 | 2,5-dimethyl-3-methoxypyrazine |

**Table S2.** Number of species in which pyrazines have been recorded across ontogeny stages. Species are omitted where there was no a clear (e.g. sex of the adult) of the studied individuals in the source. See Table S1 for identity of numbered pyrazines.

| **Order** | **Stage/sex** | **Species** | **Pyrazine number** |
| --- | --- | --- | --- |
| Phasmatodea | Adults | 1 | 12,16,17 |
|  | Male | – | – |
|  | Female | 1 | 12,16,17 |
|  | Nymphs | – | – |
|  | Egg | – | – |
| Orthoptera | Adults | 3 | 1,3,4,73 |
|  | Male | 2 | 1,3,73 |
|  | Female | 2 | 1,3,73 |
|  | Nymphs | 1 | 3 |
|  | Egg | – | – |
| Hemiptera | Adults | 8 | 3,5,73,74,75 |
|  | Male | 6 | 5,73,74,75 |
|  | Female | 6 | 5,73,74,75 |
|  | Nymphs | 1 | 73,74,75 |
|  | Egg | – | – |
| Lepidoptera | Adults | 30 | 72,73,74 |
|  | Male | 4 | 72,73,74 |
|  | Female | 1 | 73,74 |
|  | Pupae | 3 | 72,73,74 |
|  | Larvae | 5 | 72,73,74 |
|  | Egg | – | – |
| Coleoptera | Adults | 27 | 71,72,73,74,75 |
|  | Male | 4 | 72,73,74 |
|  | Female | 3 | 72,73,74 |
|  | Pupae | 1 | 72,73,74 |
|  | Larvae | 1 | 72,73,74 |
|  | Egg | 1 | 72,73,74 |
| Diptera | Adults | 9 | 1,3,4,5,6,14,22,23,25,41,55 |
|  | Male | 9 | 1,3,4,5,6,14,22,23,25,41,55 |
|  | Female | 1 | 5,22 |
|  | Pupae | – | – |
|  | Larvae | – | – |
|  | Egg | – | – |
| Hymenoptera | Adults | 144 | 1–24,26–40,42–54,56–70 |
|  | Worker | 101 | 1–17,19–24,26–40,44–46,56–64,66–70 |
|  | Gyres | 3 | 12,16,17,21,56 |
|  | Male | 20 | 5–8,12,14–18,65 |
|  | Female | 24 | 5–9,12,15,17,20,21,42 |
|  | Queen | – | – |
|  | Pupae | 1 | 6 |
|  | Larvae | – | – |

**Table S3.** Anatomical sites in which the presence of pyrazines has been detected across insects. See Table S1 for identity of numbered pyrazines.

| **Order** | **Site** | **Species** | **Pyrazine number** |
| --- | --- | --- | --- |
| Phasmatodea | Prothorax | 1 | 12,16,17 |
| Orthoptera | Whole body | 2 | 73 |
|  | Abdomen gland | 1 | 1,3,4 |
|  | Faecal | 1 | 3 |
| Hemiptera | Whole body | 2 | 73 |
|  | Haemolymph | 6 | 5,73,74,75 |
|  | Faecal | 1 | 3 |
| Lepidoptera | Whole body | 15 | 72,73,74 |
|  | Clasper scent gland | 1 | 72,73,74 |
|  | Prothorax | 1 | 73,74 |
|  | Abdominal gland | 2 | 72,74 |
| Coleoptera | Whole body | 27 | 71,72,73,74,75 |
|  | Gut | 1 | 72,73,74 |
| Diptera | Salivary gland | 1 | 3,4,5,14 |
|  | Abdomen | 8 | 1,3,4,5,6,22,23,25,41 |
|  | Rectal | 6 | 1,3,4,5,6,22,23,25,41 |
|  | Gut | 1 | 5,22 |
| Hymenoptera | Whole body | 2 | 3,5,45,46 |
|  | Head | 27 | 5,7–10,12,16–19,24,36,42,43,  47–54,56–65 |
|  | Mandibular gland | 48 | 5–8,10–17,19–21,29–36,  39,40,42,45,46,56,57,62,  66–70 |
|  | Gaster |  |  |
|  | Venom/poison gland | 37 | 1–6,9,22,23,37,38 |
|  | Dufour’s gland | 3 | 4,5,26,27 |

**Fig. S1.** Chemical structures of pyrazine compounds detected within insects.

1: Methyl-pyrazine

2: 2,6-dimethylpyrazine

3: 2,5-dimethylpyrazine

4: 2,5-dimethyl-3-methylpyrazine

5: 2,5-dimethyl-3-ethylpyrazine

6: 2,6-dimethyl-3-ethylpyrazine

7: 2,5-dimethyl-3-*n*-propylpyrazine

8: 2,6-dimethyl-3-*n*-propylpyrazine

9: 2,3-dimethyl-5-isobutylpyrazine

10: 2,5-dimethyl-3-*sec*-butylpyrazine

11: 2,6-dimethyl-3-*sec*-butylpyrazine

12: 2,5-dimethyl-3-isobutylpyrazine

13: 2,6-dimethyl-3-isobutylpyrazine

14: 2,5-dimethyl-3-*n*-butylpyrazine


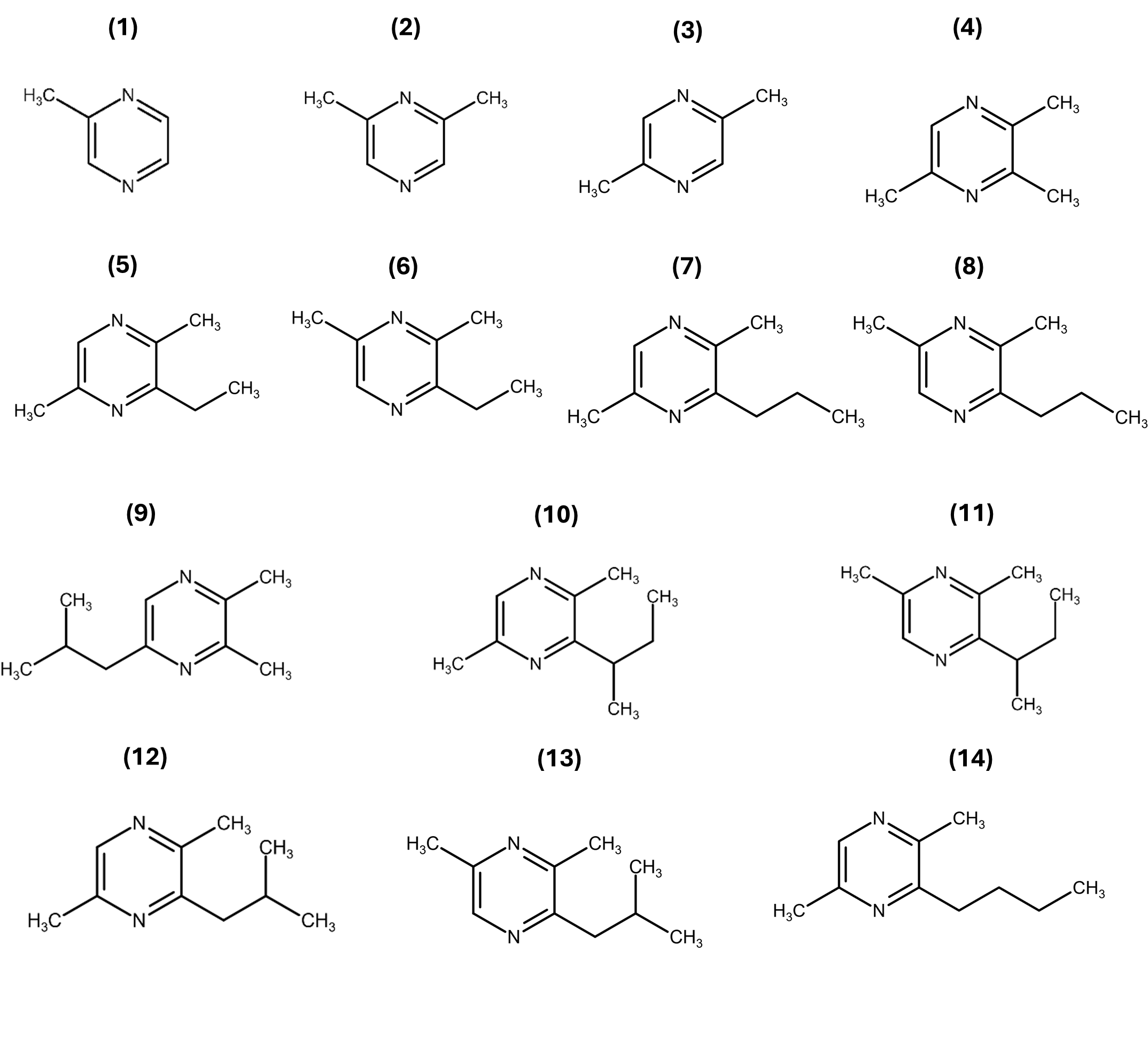


*Fig. S1 continued…*

15: 2,6-dimethyl-3-*n*-butylpyrazine

16: 2,5-dimethyl-3-(2-methylbutyl)-pyrazine

17: 2,5-dimethyl-3-isopentylpyrazine

18: 2,6-dimethyl-3-isopentylpyrazine

19: 2,5-dimethyl-3-*n*-pentylpyrazine

20: 2,6-dimethyl-3-*n*-pentylpyrazine

21: 2,6-dimethyl-3-*n*-hexylpyrazine

22: 2-ethyl-3,5,6-trimethylpyrazine

23: 2,5-dimethyl-3,6-dimethylpyrazine

24: 2,5-dimethyl-3,6-diisopentylpyrazine

25: 3,6-dihydro-2,5-dimethylpyrazine

26: 3-ethyl-2,5-dimethyl-6-propylpyrazine


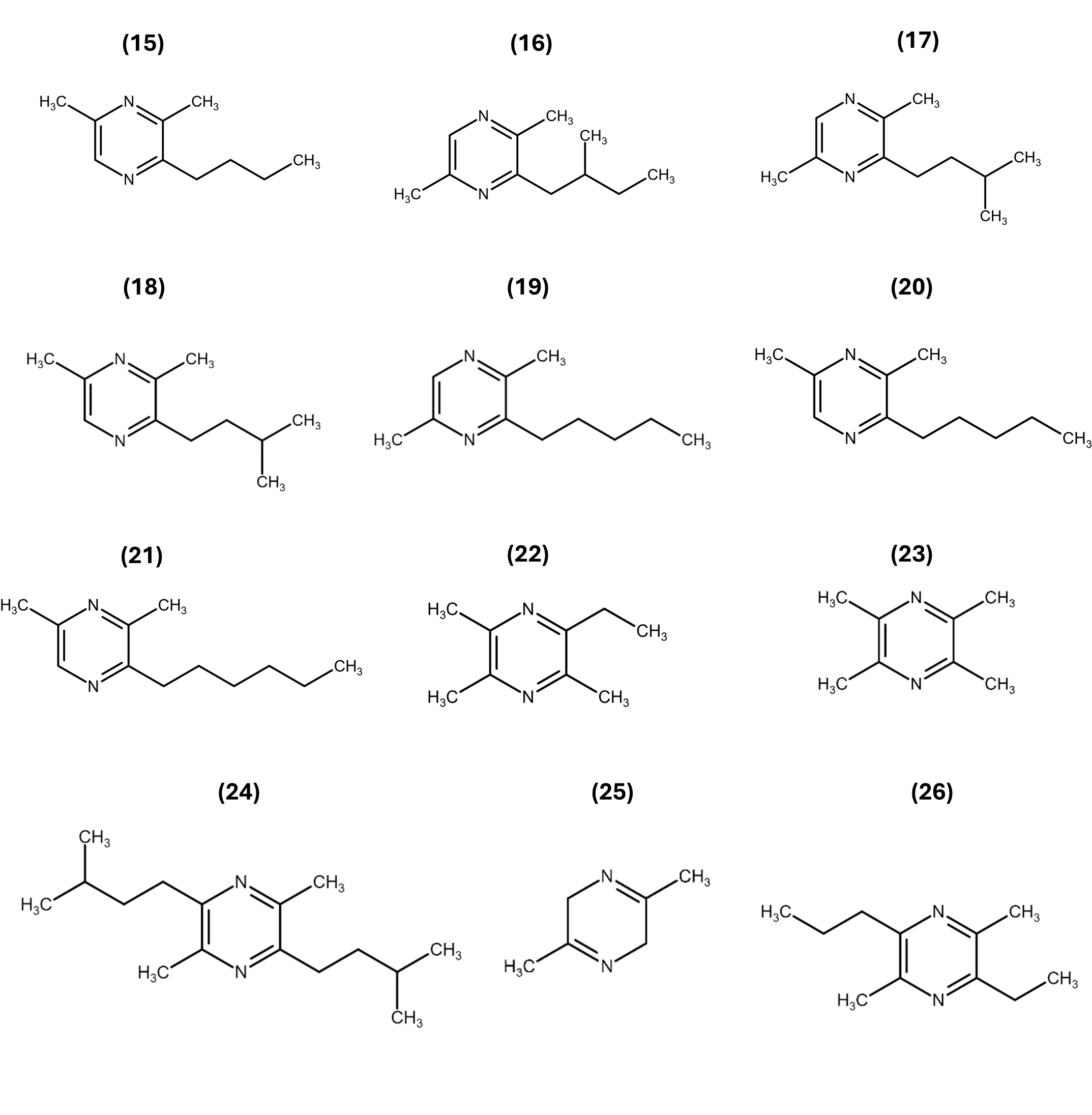


*Fig. S1 continued…*

27: 3-ethyl-2,5-dimethyl-6-isopropylpyrazine

28: 3-ethyl-2,5-dimethyl-6-butylpyrazine

29: 2,5-dimethyl-3-(2-methylhexyl)-pyrazine

30: 2,5-dimethyl-3-*n*-hexylpyrazine

31: 2,5-dimethyl-3-isohexylpyrazine

32: 2,5-dimethyl-3-*n*-heptylpyrazine

33: 2,5-dimethyl-3-isoheptylpyrazine

34: 2,5-dimethyl-3-*n*-octylpyrazine

35: 2,5-dimethyl-3-isooctylpyrazine

36: 3-methyl-2-(2-methylbutyl)-pyrazine

37: 2,3-dimethyl-5-(2-methylbutyl)-pyrazine

38: 2,3-dimethyl-5-(3-methylbutyl)-pyrazine


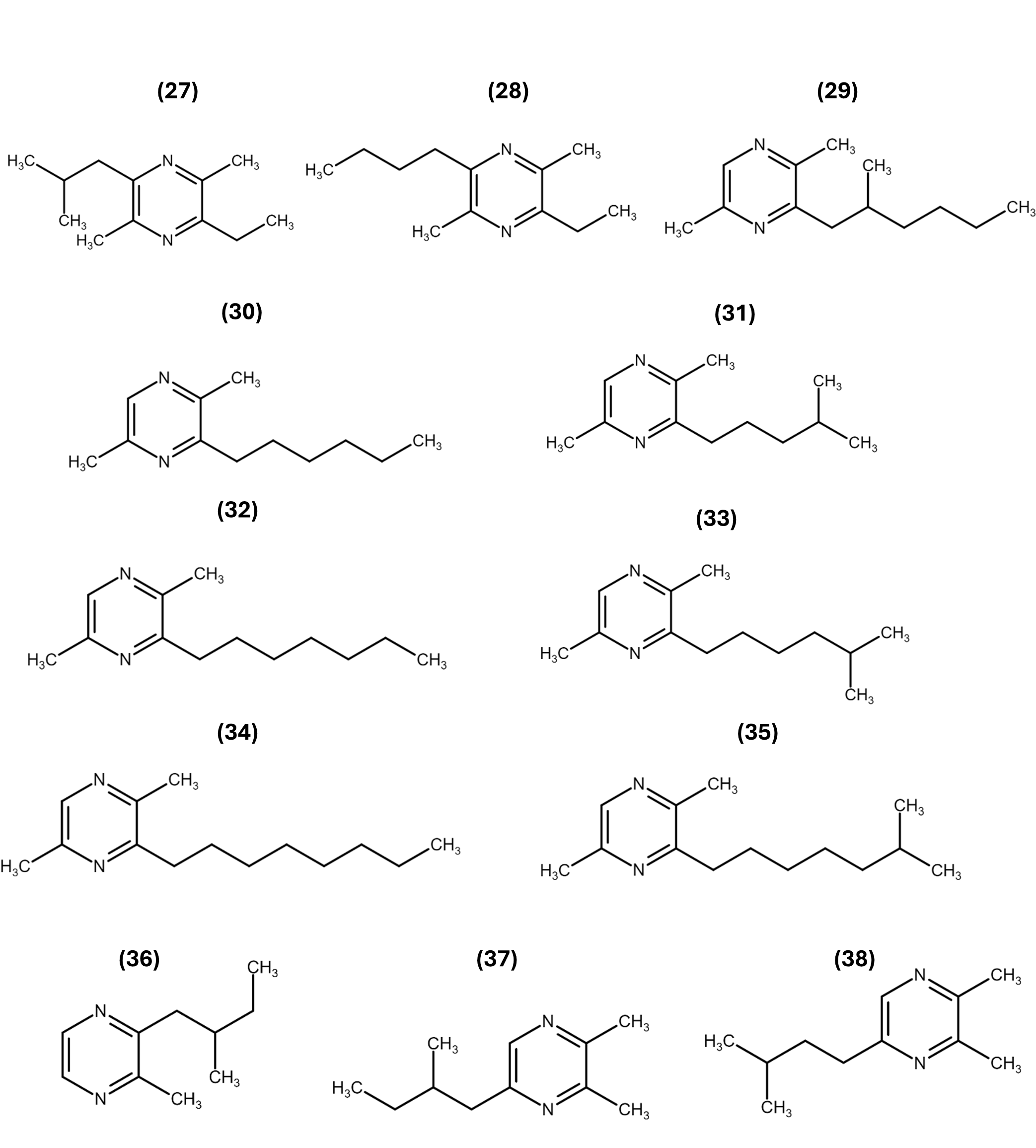


*Fig. S1 continued…*

39: 2,6-dimethyl-3-*n*-heptylpyrazine

40: 2,6-dimethyl-3-isohexylpyrazine

41: 2-methyl-6-vinyl-pyrazine

42: 2,5-dimethyl-E-3-styryl-pyrazine

43: 2,5-dimethyl-Z-3-styryl-pyrazine

44: 2,5-dimethyl-3-citronellyl-pyrazine

45: 5-methyl-3-*n*-propyl-2-(E-1-butenyl)-pyrazine

46: 5-methyl-3-*n*-propyl-2-(Z-1-butenyl)-pyrazine

47: 5-methyl-3-isopentyl-2-(E-3-methylpent-1-enyl)-pyrazine

48: 5-methyl-3-(2-methylbutyl)-2-(E-3-methylpent-1-enyl)-pyrazine

49: 5-methyl-3-isopentyl-2-(Z-3-methylpent-1-enyl)-pyrazine

50: 5-methyl-3-(2-methylbutyl)-2-(Z-3-methylpent-1-enyl)-pyrazine


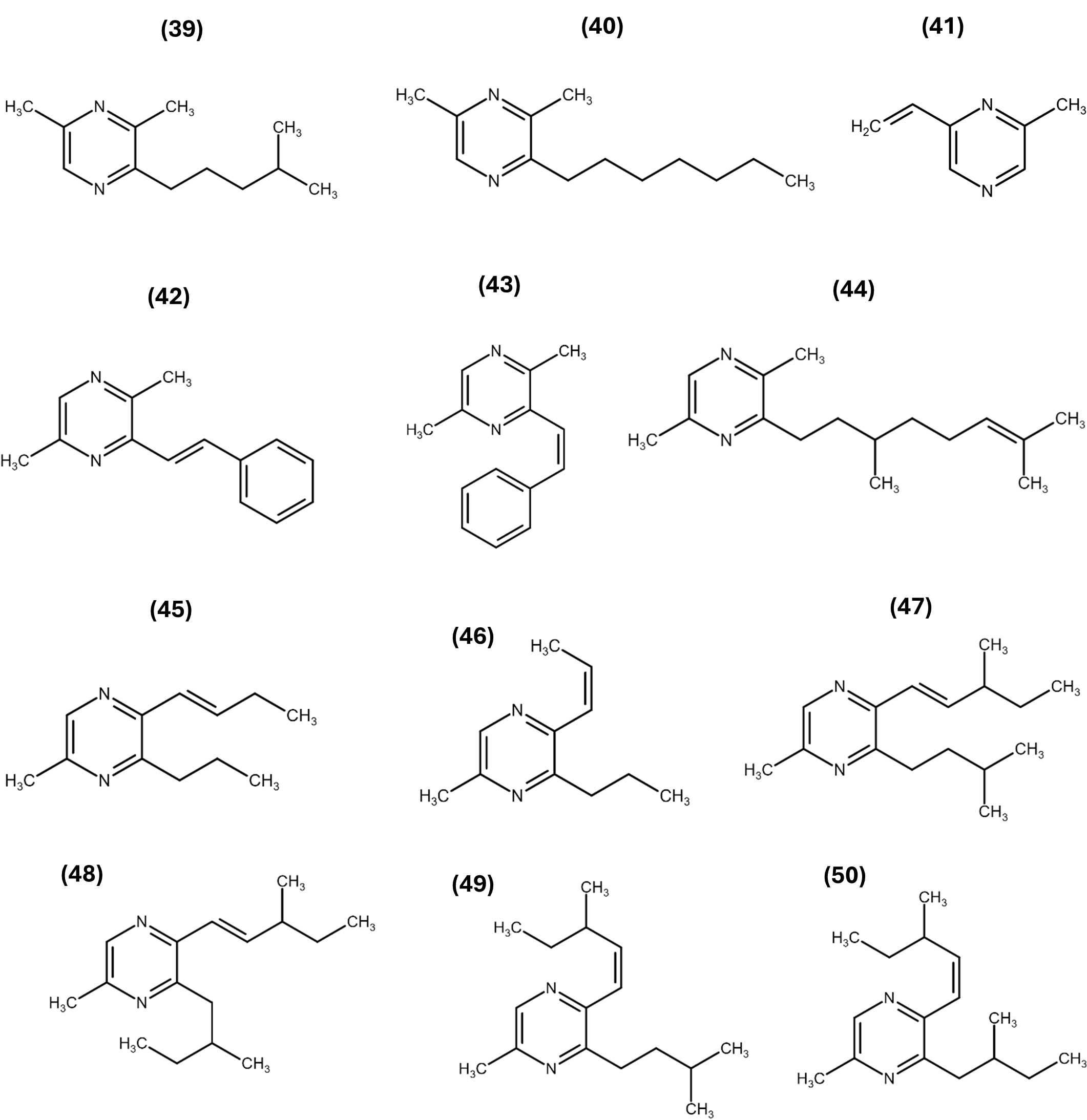


*Fig. S1 continued…*

51: 5-methyl-3-isopentyl-2(E-3-methylbuten-1-enyl)-pyrazine

52: 5-methyl-3-(2-methylbutyl)-2-(E-3-methylbut-1-enyl)-pyrazine

53: 5-methyl-3-(2-methylbutyl)-2-(Z-3-methylbut-1-enyl)-pyrazine

54: 5-methyl-3-isopentyl-2-(Z-3-methylbuten-1-enyl)-pyrazine

55: 2,5-dimethyl-3-vinylpyrazine

56: 2,5-dimethyl-3-isopentyl-6-(E-isopent-1-enyl)-pyrazine

57: 2,5-dimethyl-3-isopentyl-6-(Z-isopent-1-enyl)-pyrazine

58: 2,5-dimethyl-3-isopentyl-6-(isopent-2-enyl)-pyrazine

59: 2,5-dimethyl-3,6-di(1-hydroxyisopentyl)-pyrazine

60: 2,5-dimethyl-3-(1-oxoisopentyl)-6-(1-hydroxyisopentyl)-pyrazine


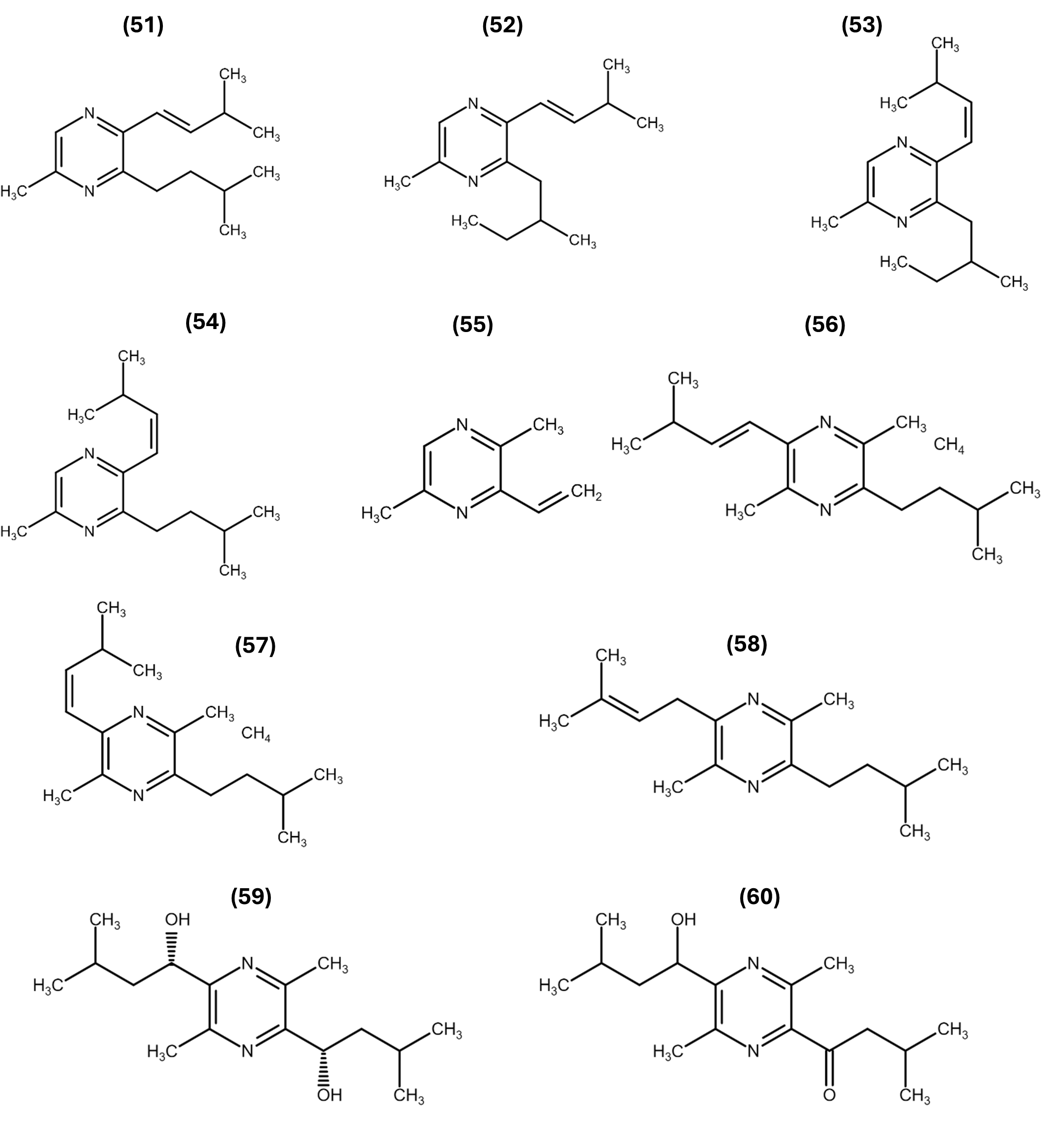


*Fig. S1 continued…*

61: 2,5-dimethyl-3,6-di(1-oxoisopentyl)-pyrazine

62: 2,5-dimethyl-3-isopentyl-6-(1-hydroxyisopentyl)-pyrazine

63: 2,5-dimethyl-3-(1-oxoisopentyl)-6-(1-oxoisopent-2-enyl)-pyrazine

64: 2,5-dimethyl-3-(1-hydroxyisopentyl)-6-(1-oxoisopent-2-enyl)-pyrazine

65: 2-hydroxymethyl-3-(3-methylbutyl)-5-methylpyrazine

66: 2,5-dimethyl-3-(1-hydroxy-3-methylbutyl)-6-(2-methylpropyl)pyrazine

67: 2,5-dimethyl-3-isopentyl-6-(1-hydroxyisobutyl)-pyrazine

68: 2,5-dimethyl-3-isopentyl-6-(1-hydroxybutyl)-pyrazine


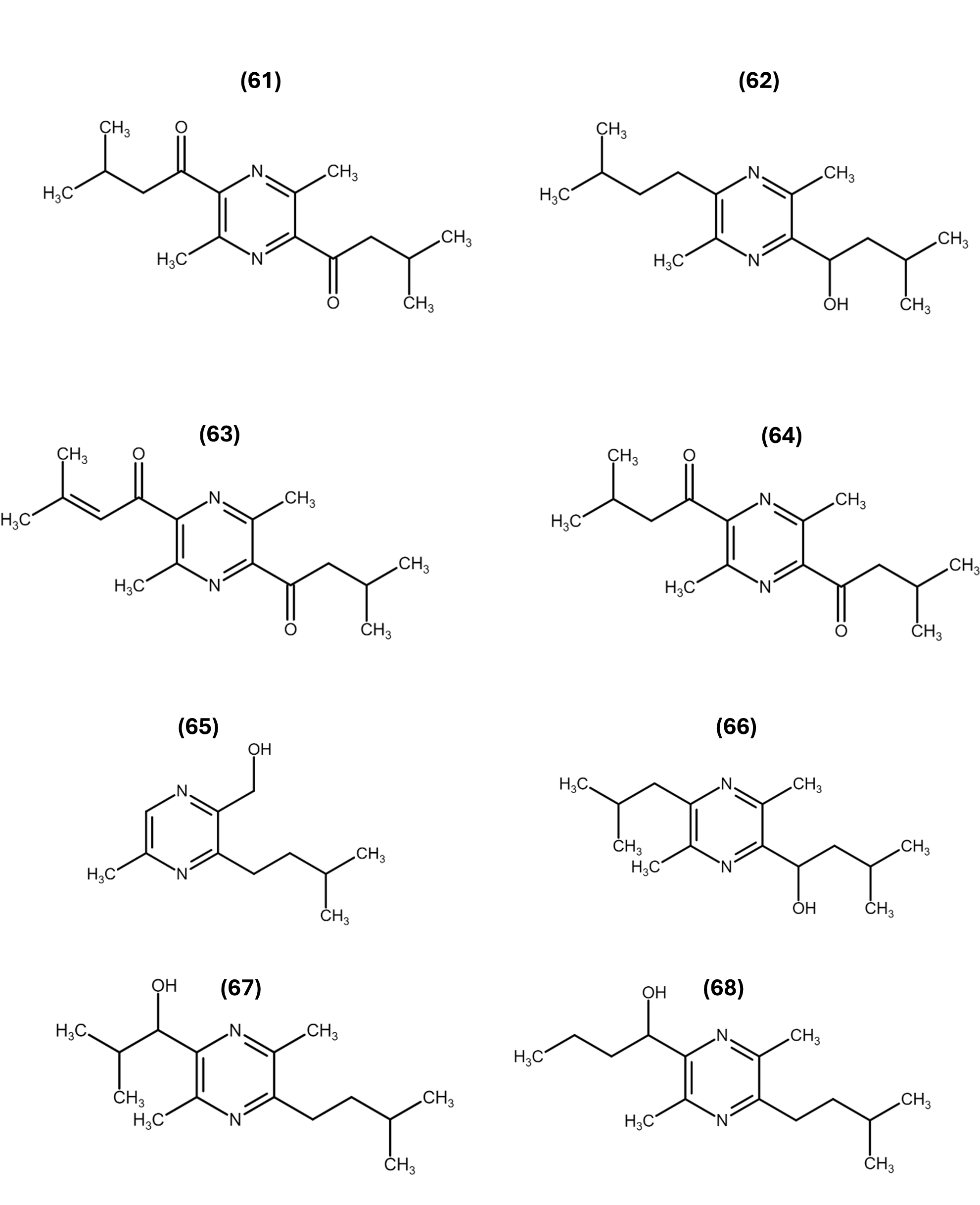


*Fig. S1 continued…*

69: 2,5-dimethyl-3-isopentyl-6-(1-hydroxypropyl)-pyrazine

70: 2,5-dimethyl-3-isobutyl-6-(1-hydroxypropyl)-pyrazine

71: 2-methoxy-3-methylpyrazine

72: 2-methoxy-3-isopropylpyrazine

73: 2-methoxy-3-*sec*-butylpyrazine

74: 2-methoxy-3-isobutylpyrazine

75: 2,5-dimethyl-3-methoxypyrazine


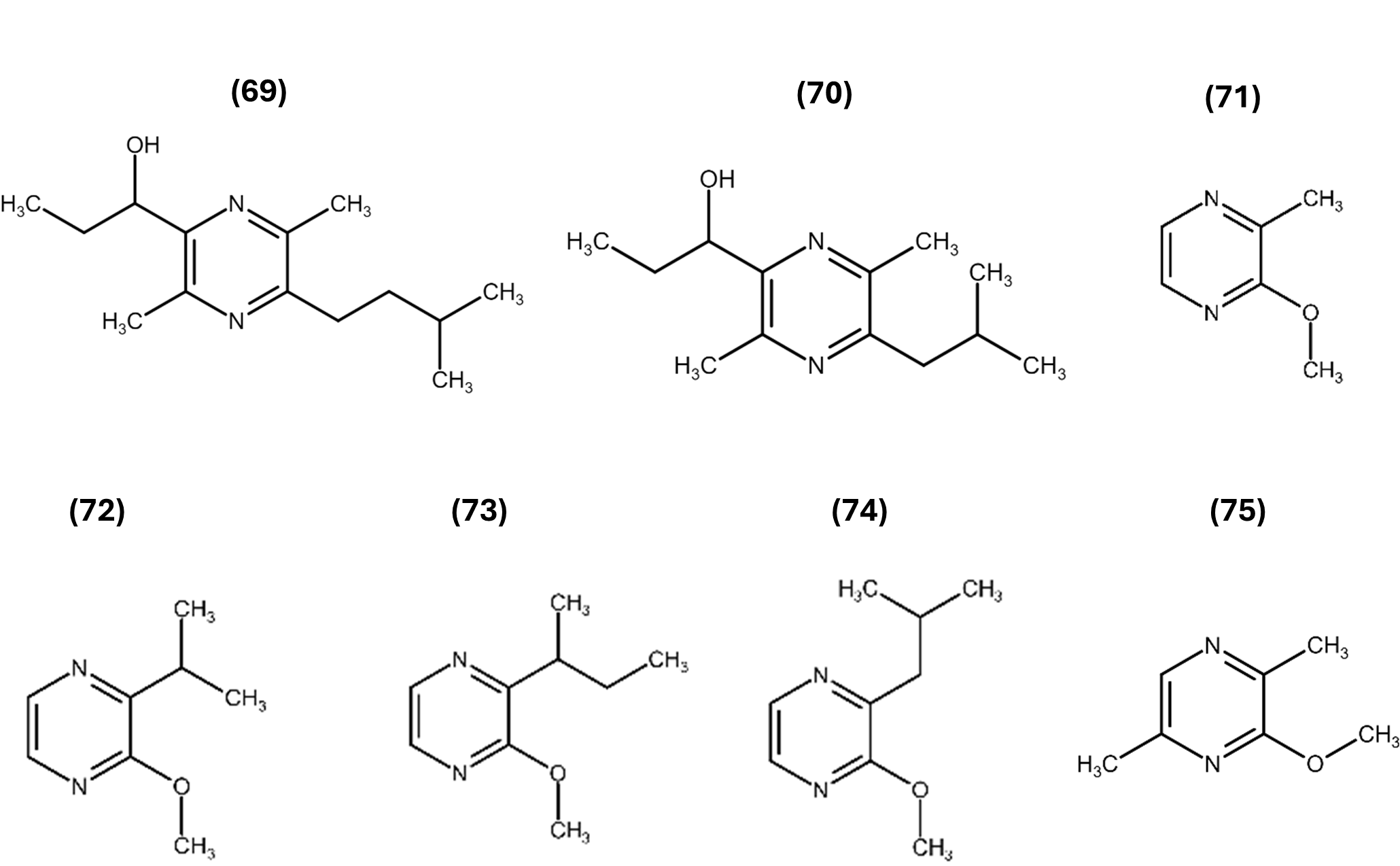

Supplement: Supplementary file 1 — Table S1. Pyrazine chemical name and the substitution groups of pyrazines detected within insects (see Table 1). Table S2. Number of species in which pyrazines have been recorded across ontogeny stages. Table S3. Anatomical sites in which the presence of pyrazines has been detected across insects. Fig. S1. Chemical structures of pyrazine compounds detected within insects. [file BRV-101-1937-s001.doc]
